# Functionalization of 2D MoS_2_ Nanosheets with Various Metal and Metal Oxide Nanostructures: Their Properties and Application in Electrochemical Sensors

**DOI:** 10.3390/bios12060386

**Published:** 2022-06-02

**Authors:** Ntsoaki Mphuthi, Lucky Sikhwivhilu, Suprakas Sinha Ray

**Affiliations:** 1 DSI-Mintek Nanotechnology Innovation Centre, Randburg 2125, South Africa; ntsoakim@mintek.co.za; 2 Department of Chemical Sciences, University of Johannesburg, Doornfontein 2028, South Africa; 3 Department of Chemistry, Faculty of Science, Engineering and Agriculture, University of Venda, Private Bag X5050, Thohoyandou 0950, South Africa; 4 Centre for Nanostructures and Advanced Materials, DSI-CSIR Nanotechnology Innovation Centre, Council for Scientific Industrial Research, Pretoria 0001, South Africa

**Keywords:** molybdenum disulfide, functionalization, metal and metal oxide nanostructures, electrochemical sensors

## Abstract

Two-dimensional transition metal dichalcogenides (2D TMDs) have gained considerable attention due to their distinctive properties and broad range of possible applications. One of the most widely studied transition metal dichalcogenides is molybdenum disulfide (MoS_2_). The 2D MoS_2_ nanosheets have unique and complementary properties to those of graphene, rendering them ideal electrode materials that could potentially lead to significant benefits in many electrochemical applications. These properties include tunable bandgaps, large surface areas, relatively high electron mobilities, and good optical and catalytic characteristics. Although the use of 2D MoS_2_ nanosheets offers several advantages and excellent properties, surface functionalization of 2D MoS_2_ is a potential route for further enhancing their properties and adding extra functionalities to the surface of the fabricated sensor. The functionalization of the material with various metal and metal oxide nanostructures has a significant impact on its overall electrochemical performance, improving various sensing parameters, such as selectivity, sensitivity, and stability. In this review, different methods of preparing 2D-layered MoS_2_ nanomaterials, followed by different surface functionalization methods of these nanomaterials, are explored and discussed. Finally, the structure–properties relationship and electrochemical sensor applications over the last ten years are discussed. Emphasis is placed on the performance of 2D MoS_2_ with respect to the performance of electrochemical sensors, thereby giving new insights into this unique material and providing a foundation for researchers of different disciplines who are interested in advancing the development of MoS_2_-based sensors.

## 1. Introduction 

Layered materials have been studied for several years. Each layered material, when thinned to its physical limit, reveals novel properties different from its bulk counterpart. Therefore, at the physical limit, these materials are referred to as two-dimensional (2D) materials [1]. These 2D materials have received great attention due to the affluence of unusual physical characteristics that occur when charge and heat transports are confined to a plane [2]. Graphene is the most widely studied 2D material because of its exceptional properties. It is composed of a single layer of carbon atoms arranged in a 2D honeycomb lattice [3]. It is a fundamental building block for a range of well-known carbon materials such as three-dimensional (3D) graphite, one-dimensional (1D) carbon nanotubes, and zero-dimensional (0D) fullerene [3]. Since its isolation, it has inspired thoughtful research on other 2D materials. These materials are useful building blocks that can be restacked and incorporated into composites for a wide range of applications. Other than graphene, recent efforts have focused on other 2D materials (ranging from conductors to insulators). These materials include transition metal dichalcogenides (TMDs) as semiconductors involving a general chemical formula of MX_2_, where M is a transition metal atom (M = Mo, W, Ti, Zr, Ta, Nb, Re, Ni, or V) and X is a chalcogenides atom (X = S, Se, Te), as well as black phosphorus (BP), transition metal oxides, and hexagonal boron nitride (hBN) as an insulator [3,4,5]. TMDs are now the key focus of many researchers because of their unique properties. Exfoliated 2D TMDs have properties that are complementary to (but yet distinct from) those of graphene, and they also have the advantages of tunable bandgaps ranging from 1–2 eV [4,6,7]. One of the most widely explored transition metal dichalcogenides is molybdenum disulfide (MoS_2_) [8,9,10]. The electrical and optical properties of MoS_2_ are layer-dependent. For instance, bulk MoS_2_ is an n-type semiconductor with an indirect bandgap of about 1.3 eV, while a monolayer MoS_2_ has a direct bandgap of 1.8 eV [6,11]. Moreover, monolayer MoS_2_ exhibits carrier mobility of 200 cm^2^V^−1^s^−1^ at room temperature with a high on/off current ratio of ~10^8^ [12]. MoS_2_ has a layered crystal structure formed by stacking covalently bound S-Mo-S monolayers through weak van der Waals interactions [13,14]. The van der Waals interlayer interaction between these monolayers allows for their separation leading to crystal exfoliation with approximately 0.65 nm of thickness (6.5 Å) [12,15].

As a semiconducting analog of graphene, this material has recently been reported as promising in the application of sensors [13], photodetectors [16], transistors [12], flexible electronics [17], and fuel cells [18]. Although the use of MoS_2_ nanosheets offers many advantages, poor conductivity (due to the large bandgaps) considerably limits their practical applications in sensors. Moreover, it has been reported that pristine MoS_2_ nanosheets suffer from gradual degradation at ambient conditions, which are triggered by surface contamination and significant adsorption of oxygen in an air environment. This leads to instability and decreased electrical properties and sensing abilities [19,20]. Therefore, to obtain satisfactory results, pristine MoS_2_ nanosheets need to be operated in an inert atmosphere. To overcome these limitations and extend the potential application of MoS_2_ nanosheets, various approaches have been attempted, such as the substitution of the transition metal of MoS_2_ with other elements [21], molecular physisorption of organic materials [22], changing interfacial chemistry [23,24], solution-based chemical doping [25], and promoting structural phase transition (from semiconducting 2H phase to metallic 1T phase) [26,27]. However, some of these methods are complicated and time-consuming; some result in structural defects, reduced mobility, and unstable composites [28]. Furthermore, researchers noted that TMDs have a habit of being inert to chemical functionalization. Chalcogen atoms in the basal plane of TMD nanosheets are saturated and, therefore, are not highly reactive, whereas the metal sites in TMDs are embedded beneath the chalcogen layer, all but eliminating them from being useful for functionalization [29]. Despite this, it is necessary to functionalize MoS_2_ with chemical moieties, which can enable its interface with other nano- or micro-structures [15]. The synergetic effects resulting from MoS_2_ composites can produce enhanced properties or improved performances. The large surface-to-volume ratio of 2D MoS_2_ nanosheets presents an opportunity for the effective surface functionalization of the material with metal and metal oxide nanostructures (NSs). Compared with previously described methods of functionalization, interfacing 2D MoS_2_ nanosheets with metal-based NSs is more simply carried out and offers an additional path for controlling the thermal, catalytic, magnetic, optical, and electrical properties. Based on theoretical calculations, it has been reported that the effective adsorption of different metal atoms on 2D MoS_2_ is related to the number of d-electrons, which can also effectively modulate the band structures of the material [30]. Most metal-based NSs have good stability and are resistant to environmental deterioration and oxidation, which make them ideal for the fabrication of nanocomposites. However, it is worth noting that the structures and properties of nanocomposites are dependent on the conditions of the composite synthesis and the control of the decorated location, morphology, and number density of the NSs [31]. Recently, metal and metal oxide nanocomposites have received a great deal of attention because of their excellent physical and chemical properties, which expanded their application in sensors [20,31], electrocatalysis [32,33], and optoelectronics [34]. Thus, it can be expected that 2D MoS_2_ nanosheets decorated with metal-based NSs could potentially extend their applications as novel nanomaterials in sensors. For instance, 2D MoS_2_ functionalized with metal and metal oxide NSs, such as nickel (Ni) [35], gold (Au) [36], and tricobalt tetraoxide (Co_3_O_4_) [37], to name a few, was reported to exhibit excellent catalytic sensing properties. The MoS_2_-Ni nanocomposite demonstrated good reproducibility and excellent sensitivity toward glucose detection. The reported results showed that small Ni nanoparticles (NPs) on the surface of the MoS_2_ nanosheet had more active sites, resulting in high electrocatalytic activity and a fast response time of less than 2 s. In addition, it was discovered that Ni NPs without MoS_2_ support appeared to aggregate, which was detrimental to the electrocatalytic activity of the sensor [35]. The MoS_2_-Co_3_O_4_ nanocomposites showed high sensitivity and fast response and recovery features for the detection of ammonia at room temperature. The sensing film was constructed on an interdigital electrode substrate using layer-by-layer self-assembly of MoS_2_ nanosheets and Co_3_O_4_ nanorods. The layer-by-layer self-assembly not only efficiently prevented agglomeration but also provided many more active catalytic sites on p-type Co_3_O_4_ nanorods toward ammonia. The results indicated that the fundamental sensing mechanisms of the MoS_2_-Co_3_O_4_ nanocomposite towards ammonia were attributed to the layered nanostructure, synergistic effects, and p-n heterojunction depletion layer formed at the interface of n-type MoS_2_ and p-type Co_3_O_4_ [37]. These 2D-MoS_2_/metal-NSs composite sensor films showed significant improvements in sensitivity in comparison with the 2D MoS_2_ and metal/metal oxide nanostructure counterparts, suggesting that the large surface areas, high conductivity, and improved biocompatibility played significant roles in the resulting sensing outcome. Su et al. [10], also reported that MoS_2_ stabilizes metallic NPs, such as platinum (Pt), Au, silver (Ag), and lead (Pd) to form hierarchical nanocomposites, and such 2D-MoS_2_/metal-NSs composites possess the essential properties of pure metal NPs and MoS_2_ nanosheets due to their synergistic effects, making the 2D MoS_2_ and metal/metal oxide nanostructured composites exhibit excellent electrochemical properties for the fabrication of electrochemical sensors [13]. 

In the last few years, studies focused primarily on the preparation, properties, and application of pristine 2D MoS_2_ for sensor application [38,39,40,41]. However, the preparation and sensing applications of 2D MoS_2_ nanosheets have recently gone through numerous new developments by functionalizing 2D MoS_2_ with different metal-based NSs. This upcoming trend carries the potential for expanding the application of 2D MoS_2_ nanosheets as novel nanomaterials in electrochemical sensors. This is done by enhancing the material’s electrocatalytic ability and increasing the active surface area, conductivity, functionality, and rapid heterogeneous transfer of electrons. The objective of this review was therefore to provide a critical overview of the recent advantages and limitations in the use of functionalized 2D MoS_2_ nanosheets. Studies show that 2D MoS_2_ nanosheets functionalized with metal-based NSs have additional benefits and unique properties that are distinctive from their pristine counterpart. Our main emphasis is on the preparation and functionalization method, their properties, and their applications in electrochemical sensors.

## 2. Synthesis of MoS_2_ Nanosheets

Different methods for synthesizing 2D MoS_2_ nanosheets have been proposed; these methods have also been used for tuning 2D MoS_2_ electronic band structures. So this creates an opportunity to find new methods that are appropriate for sensing applications. Lately, MoS_2_ nanosheets have been produced by either mechanical or chemical methods. In a standard mechanical exfoliation procedure, sufficient thin MoS_2_ crystals are first peeled off from their bulk crystals, layer-by-layer, using adhesive Scotch tape and exerting normal force. By repeating this process multiple times, the bulk MoS_2_ becomes thinner until it is reduced to a single sheet of MoS_2_. The cleaved thin crystals are detached with devices, such as plastic tweezers, and are adsorbed onto the target substrate [42]. While this method can yield pristine and high-quality 2D MoS_2_ nanosheets, poor scalability limits its practical application in general.

**Figure 1:**
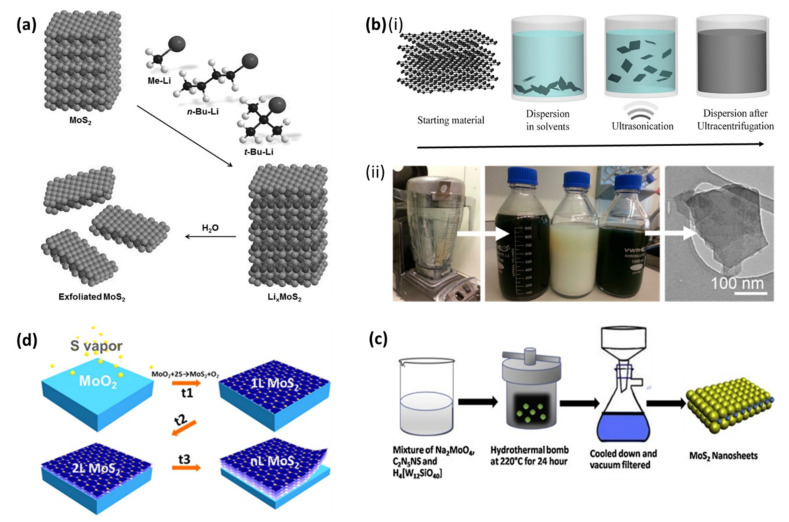
Schematic diagrams of (**a**) lithium intercalation exfoliation [44], (**b**) (i) exfoliation by sonication [48], (ii) exfoliation by high shear rate using a basic kitchen blender [50], (**c**) hydrothermal synthesis [54], (**d**) CVD method [56]. Figures reproduced with permission from Ref. [44], © 2014 WILEY-VCH Verlag GmbH & Co. KGaA, Weinheim, Germany, Refs. [48,50] copyright © 2015, American Chemical Society, Washington, DC, USA, Ref. [54] © 2018 Elsevier B.V., Amsterdam, The Netherlands. All rights reserved, Ref. [56] copyright © 2013, American Chemical Society.

Chemical methods include:

(i) lithium (Li) intercalation exfoliation, which involves an initial 48 h intercalation of Li ions between bulk MoS_2_ in an inert gas atmosphere followed by a spontaneous ultrasonic-assisted exfoliation of the Li-intercalated MoS_2_, which occurs through water reaction, as shown in Figure 1a [43,44]. During the process, bulk MoS_2_ is treated with an n-butyllithium solution to produce the intercalation product Li_x_MoS_2_. The reaction process is caused by the electron transfer from n-butyllithium to MoS_2_ layers, which absorbs Li^+^ between the layers to balance the charge. When the intercalate, Li_x_MoS_2_, comes into contact with water, it reacts with the intercalated lithium, creating LiOH and H_2_ gas. As a result, negatively-charged nanosheets repel each other, increasing interlayer distances and weakening van der Waals forces, resulting in a colloidal dispersion of (nearly) entire single layers of MoS_2_ [45]. The lithium intercalation reaction converts MoS_2_ from the semiconductive 2H phase hexagonal structure to the metallic trigonal 1T phase [46], although the 1T phase MoS_2_ is not suitable for optoelectronic devices, it is quite desirable for electrochemical catalysis.
(ii) Liquid-phase exfoliation (Figure 1b) involves exfoliation by sonication [47,48] or exposure of bulk MoS_2_ to high shear rates using either a rotor–stator high shear mixer or a basic kitchen blender [49,50]. The bulk MoS_2_ is exfoliated in the presence of stabilizing liquids, such as suitable solvents [47], surfactant solutions [50], or polymers [51]. Sonication-assisted exfoliation is triggered by hydrodynamic shear forces associated with cavitation, which is the formation, growth, and collapse of voids or bubbles in liquids due to pressure changes [48]. Following exfoliation, the inter-sheet attractive forces and interfacial tension between the sheets and the surrounding liquid are reduced, resulting in the dispersion of a single layer [48]. When using a high shear mixer or blender, exfoliation occurs as a result of revolving rotor–stator blades producing extremely high shear rates [49,50]. The shear rate generates full turbulence, resulting in viscous shear forces. The presence of dominant viscous shear forces increases collision and cavitation between bulk materials, leading to exfoliation [49,50]. During this process, the sheets are coated with an appropriate surfactant/solvent and stabilized by repulsive inter-sheet interactions [50]. This approach results in defect-free single or multilayered nanosheets that are stabilized against aggregation by liquid interaction. The liquid-phase exfoliation method, due to its simplicity and scalability potential, has an advantage over the lithium exfoliation method; it is also not an air-sensitive process and it does not require chemical reactions; thus, it provides high crystallinity for synthesized MoS_2_ nanosheets [52].
(iii) Hydrothermal/solvothermal synthesis (Figure 1c) is essentially where the chemical reaction takes place in a closed system (autoclave) in which the solvent temperature is raised to its critical point (>200 °C) by heating concurrently with autogenous pressure [53]. This approach involves direct crystallization from solutions, which often include crystal nucleation and subsequent growth [53]. The sample is rapidly precipitated from the reaction solution during the synthesis process, allowing for controlled homogeneity, as well as control over aging, particle size, and morphology [54]. The solvothermal synthesis is similar to the hydrothermal synthesis, except that in the synthetic method organic solvents are used instead of water [55]. MoS_2_ nanosheets with good crystalline structure and morphology are produced by optimization of hydrothermal temperature and reaction time [54].
(iv) Chemical vapor deposition (CVD) (Figure 1d) has been used to synthesize high-quality graphene, and it was recently adopted for the synthesis of MoS_2_ nanosheets. In a standard CVD procedure, molybdenum trioxide (MoO_3_) and sulfur powders are common precursors used for the deposition of MoS_2_ films on a silicon/silicon dioxide (Si/SiO_2_) substrate; upon heating, MoO_3_ reacts with sulfur vapor in the gas phase at a high temperature (>650 °C) to give MoS_2_ layers in a reducing atmosphere under ambient pressure [56]. The CVD process is capable of producing nanosheets of good quality with scalable size, controllable thickness, and excellent electronic properties. However, it is more difficult to obtain crystalline 2D MoS_2_ nanosheets with a controlled number of layers by CVD as compared to graphene, because the structure, thickness, and crystallinity of graphene could be well controlled by an effective catalyst design but there is no catalyst involved in the growth of 2D MoS_2_ nanosheets [56,57].

The important thing to realize is that due to the nature of the exfoliation method, many imperfections, such as surface defects, may occur. The usual defect positions that occur are sulfur vacancies on the surface or edges [58,59]; due to these defects, 2D MoS_2_ nanosheets have low charge carrier mobility and density [59]. These defects were recognized as possible synthetic targets for the functionalization and modification of MoS_2_ surfaces for various additional applications [58]. The functionalization of MoS_2_ is important to adjust or add the required properties to the material, which can be utilized in the fabrication of sensors and catalysts. This is an advantage given that 2D MoS_2_ offers abundant exposed edges that are known as the root of the catalytic activity [60,61].

## 3. Functionalization of MoS_2_ Nanosheets with Metal and Metal Oxide Nanostructures

Metal and metal oxide NSs are known for their exceptional electrical and catalytic properties; hence, they are the most widely used nanomaterials. The application of these nanomaterials to medicine [62], energy storage, catalysts, sensors [63,64,65], and electronics [66] has led research into the development of synthetic pathways toward the formation of nanocomposites. The integration of these NSs on MoS_2_ can effectively exploit the distinct qualities of both materials, which have already attracted considerable attention in the sensing field. The metal-based NSs can be distributed and significantly improved by the effective support matrix of MoS_2_ nanosheets, where the synergistic effects of metal-based NSs and MoS_2_ nanosheets can lead to greater catalytic efficiency and conductivity than plain MoS_2_. Several factors play significant roles in the properties of metal and metal oxide NSs, such as crystal structure, size, shape, morphology, and surface chemistry [67]. Thus, structuring methods have been applied to obtain different sizes and shapes of NSs with promising properties, such as high sensitivity, faster electron transfer kinetics, low background currents, high surface areas, and high current densities [68]. Metal and metal oxide NSs have been used to modify electrodes for use as electrocatalysts in sensors; hence, they play significant roles in diagnostic devices. The 2D-MoS_2_/metal-NS composites utilize the optimum availability of the nanoscale surface area for electron transfer, and also enable mass transport of the reactants to the electroactive focal point on the electrode surface, resulting in a significantly improved electrochemical response [69,70]. 

The decoration of MoS_2_ nanosheets with metal-based NSs is commonly carried out in two different ways: in situ or ex situ functionalization. These functionalization methods can be achieved by post immobilization of NSs on MoS_2_ [69], hydro/solvothermal reaction [70], electrodeposition [13], or the chemical reduction method [35]. In these functionalization methods, the growth control of NSs on MoS_2_ nanosheets is a prerequisite for tuning the shape, size, and morphology. This growth control however involves precise process monitoring of the experimental parameters, such as the concentration of the reactant, time and temperature of the reaction, pH solution, the surfactant used, and the type of metal salts [71,72]. For instance, in a chemical synthesis process, the size and shape of the NPs can be effectively controlled by the concentration of metal salts, reaction conditions, or the use of different surfactants. Moreover, through an electrochemical synthesis, the concentration of surfactants, growth temperature, and current density can be optimized to control the size and shape of the NPs [72]. Concerning the subject, a study was conducted where thionine (C_12_H_10_N_3_S^+^)-MoS_2_ was functionalized with AuNPs by a hydrothermal reaction for electrochemical immunosensing [73]. C_12_H_10_N_3_S^+^ was used as a reducing agent and a surfactant to tune the resulting AuNPs structures on the surface of the MoS_2_ nanosheets. The study demonstrated that various well-defined shapes of AuNPs were produced by adjusting the concentration of C_12_H_10_N_3_S^+^ while the concentration of metal salt (HAuCl_4_) remained constant. It was discovered that with an increasing concentration of C_12_H_10_N_3_S^+^, the size of the NPs increased and the shape changed from spherical, triangle, and clover-like to flower-like shape. A significant finding was that C_12_H_10_N_3_S^+^ could not facilitate the growth of AuNPs in the absence of MoS_2_ nanosheets. This indicated that C_12_H_10_N_3_S^+^ and MoS_2_ had a synergistic effect on the formation of AuNPs and the growth of AuNPs was also promoted by the effective support matrix of MoS_2_ nanosheets. The proposed mechanism was because C_12_H_10_N_3_S^+^ is an electrochemical indicator, the growth of AuNPs on the surface of MoS_2_ nanosheets could be due to the redox reaction among MoS_2_, C_12_H_10_N_3_S^+^, and AuCl_4_^−^. C_12_H_10_N_3_S^+^ was absorbed on the surface of MoS_2_ nanosheets through a π−π  interaction and an electrostatic interaction due to its planar aromatics structure. Therefore, MoS_2_/AuCl_4_^−^ and C_12_H_10_N_3_S^+^/AuCl_4_^−^ formed two redox pairs, enabling the spontaneous transfer of electrons from MoS_2_ and C_12_H_10_N_3_S^+^ to Au ions, allowing the reduction of Au ions to AuNPs on the MoS_2_ nanosheets [73,74,75].

Recently published reviews have reported on the synthesis and application of metal and metal oxide NSs [72,76]. The reviews included extensive information on the effects of structural morphology, size, and shape on the properties of NSs and their application in the field of electrochemical sensors. Interestingly, all studies clearly indicate that the shape and structural morphologies of NSs serve important roles in determining their resulting electrocatalytic properties, as the number of exposed catalytic active sites is specifically dependent on the shape of the NPs. That being said, it was also reported that a perfect shape–surface structure interaction is unrealistic and, even with a well-defined size and shape, its surface will be extremely complex. It consists of not only some ordered surface domains of different dimensions but also a defined number of defects, corners, edges, steps, and kink sites, all of which contribute to the resulting electrocatalytic activity [77]. 

Many methods for the preparation of size and shape-controlled metal-based NSs are now available in the literature. Various sizes, shapes, and structures can be synthesized, such as 0D NSs, which include structures, such as NPs, quantum dots, and nanospheres, as well as 1D NSs with high aspect ratios, such as nanorods, nanocombs, nanofibers, nanotubes, nanoneedles, nanoribbons, and nanowires. The 2D NSs are known as nanoplates, nanosheets, and nanopellets, while nanocrystals, multi-nanolayers, nanoflowers, and snowflakes are characterized as 3D NSs [76,78]. A comprehensive effort has been made to control the shapes and sizes of metal-based NSs in order to guarantee their efficient performances in electrochemical sensors. The synthesis of various forms of NSs not only enhances physical and chemical properties but also improves the biocompatibility and bioefficacy for the immobilization of biomolecules [72,76,79]. Moreover, due to the many active sites provided by particular NSs, a wide linear range and low detection limits have been achieved, resulting in NSs being ideal materials for the fabrication of biosensor devices. Table 1 summarizes the various functionalization methods of MoS_2_ nanosheets with different metal and metal oxide NSs, their resulting structural morphologies (size and shape), and their application in electrochemical sensors. The commonly used technique for making 2D-MoS_2_/metal-NSs composites is in situ chemical growth.

### 3.1. Ex situ Functionalization

Ex situ functionalization (post immobilization) requires the mixing of a MoS_2_ nanosheet solution with pre-synthesized NPs to form a composite. Even though the NPs and 2D material suspensions are independently pre-synthesized, the ratio of the two materials can be controlled with precision [110]. In this method, NPs are synthesized in advance and then attached to the MoS_2_ surface through covalent [80] or noncovalent interactions, such as van der Waals or electrostatic interactions, and hydrogen bonding [111]. The ex situ functionalization by absorption is much easier, more flexible, and ideal for commercial applications on a large scale than the in situ functionalization procedure. The deposition of metal NSs on the surface of MoS_2_ is determined by the type of bonding and, thus, the strength of interaction. In covalent functionalization, the metal-based NSs are attached to the edge and surface of MoS_2_ through covalent bonding between metal atoms of NSs and sulfur atoms of MoS_2_ [15,80]. The sulfur vacancies on MoS_2_ are created by severe defects and edges caused by the loss of atoms during the intense chemical exfoliation [112]. The vacant sulfur atoms that bind to metal atoms serve as sites for metal nuclei seeding and subsequent growth into larger NSs [15,111]. The covalent bonding of NSs usually results in a strong and stable attachment that prevents the leaching of particles. The main advantage of covalent bonding is that it uses efficient and controlled chemical interactions to avoid side reactions and prohibits undesirable formations [113]. Alternatively, metal NSs can be attached to MoS_2_ via noncovalent interactions. Electrostatic interactions are the commonly utilized noncovalent approaches in the syntheses of MoS_2_-based nanocomposites [69,81,82]. Absorption of MoS_2_ is often achieved via surfactant functional groups attached to the surface of metal and metal oxide NSs. The negatively charged MoS_2_ nanosheets are then leveraged to electrostatically interact with positively charged metal-based NSs, resulting in strong interactions and high particle loading [69,114]. Non-covalent functionalization provides the advantage of allowing metal NSs to be introduced into MoS_2_ without changing its structure, preserving the unique features of 2D MoS_2_ [110,111]. Based on the above-mentioned interactions and the nature of the attached NSs, the optical, catalytic, and electrical properties of MoS_2_ could be improved. For instance, electrostatic interactions offer more conductive interlayers for electron transfers between MoS_2_ nanosheets and absorbed metal NSs, resulting in better electronic characteristics of MoS_2_ [69]. Covalent bonding, on the other hand, offers a high density of active sites and a large surface area by the strong and stable attachment of metal NSs and induced surface defects, thereby enhancing the catalytic activity of the MoS_2_ nanosheets [115,116].

**Figure 2:**
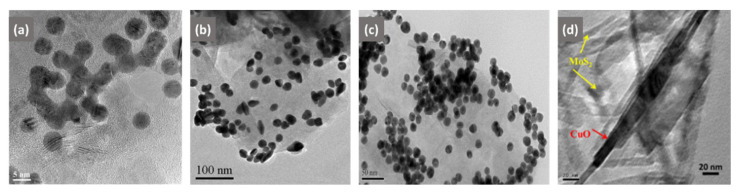
TEM images of (**a**) MoS_2_-Au [69], (**b**) TTR-MoS_2_-Au [80], (**c**) MoS_2_-PEI-Au [81], (**d**) CuO-MoS_2_ [82] nanocomposites produced by ex situ functionalization. Figures reproduced with permission from Ref. [69] copyright © 2016 Elsevier B.V., Refs. [80,81] copyright © 2017 Elsevier B.V., Ref. [82] copyright 2020 Elsevier, Ltd. and Techna Group.

The main challenge aimed at ex situ functionalization is the ability to synthesize NPs that have high dispersibility and long-term aggregation stability. The uniform distribution of NPs is mostly hindered by phase separation, segregation, and agglomeration, which deteriorate the properties of the material. The use of capping agents and surfactants helps prevent particle agglomeration and reduces any phase separation due to the material’s incompatibility. Owing to electrostatic interactions, the polar groups of surfactants can be adsorbed on the surface of NPs [117]. Surfactants can reduce particle–particle interactions and, thus, decrease the formation of agglomerates as physical attraction forces decrease [118]. However, a downside of using surfactants is that they are thermally and solvolytically unstable because of the relatively weak van der Waals forces or the hydrogen bond that binds them to the surface of NPs [117]. It remains a challenge to achieve a homogeneous dispersion of non-agglomerated NPs in the nanocomposite matrix by the typical ex situ functionalization method; hence, researchers tend to also use ultrasonication techniques to disperse NPs on 2D materials. For example, Au nanoparticles were immobilized on the MoS_2_ surface by ultrasonication for the fabrication of electrochemical biosensors [69,80]. The transmission electron microscope (TEM) image verified the immobilization of Au nanoparticles in close proximity to each other on MoS_2_ nanosheet surfaces via covalent bonding between Au atoms and sulfur atoms (Figure 2b) [80]. This was due to the direct surface affinity between the integrated structures of MoS_2_ and the Au nanocrystals. In yet another instance, an electrochemiluminescence immunosensor was fabricated based on MoS_2_-PEI-Au nanocomposites formed through the dispersion of MoS_2_-PEI into Au NPs by stirring the solution overnight (Figure 2c) [81]. Au NPs were distributed randomly on the layered surface structures of MoS_2_ nanosheets. On the other hand, the MoS_2_-CuO nanocomposite (Figure 2d) obtained by stirring the two nanomaterials for hours showed the reduced CuO nanotube aggregation [82]. The agglomeration of the NPs may appear to have had negative impacts on the properties of the nanocomposite. However, in a rare instance, agglomeration of Au NPs on the surface of MoS_2_ was discovered to be particularly favorable for enhancing the photocurrent response of MoS_2_ based on the effects of surface plasmon resonance [80]. Combined with thin layers of 2D MoS_2_, they efficiently transformed the local plasmonic enhancing effect into an electrical signal, thus improving the photoelectrochemical response of MoS_2_ [80]. Figure 2 shows the TEM images of 2D-MoS_2_/metal-NSs composites produced by the ex situ functionalization method; the NPs exhibited some agglomeration and displayed a non-uniform coverage. It should be noted that the arrangements of the NSs on the surface and within the MoS_2_ nanosheet matrix depend greatly on the functionalization process.

### 3.2. In Situ Functionalization

The in situ functionalization is the most commonly used approach and an effective way of preparing nanocomposites based on 2D MoS_2_ decorated with metal-based NSs. In this technique, the NPs are synthesized within the matrix or the surface of MoS_2_ nanosheets from metal salts that are converted into required NPs by order of reduction reactions.

This approach has the advantage of preventing particle agglomeration while improving the overall spatial distribution of NPs. The in situ functionalization has gained attraction over ex situ functionalization because it provides greater control over the material’s structure and properties, and particle size can be regulated with minimal effort. The in situ approach is more cost-effective and has an inherent benefit that the interfaces of the nanocomposite matrix are clean, resulting in good interfacial bonding. NPs are frequently produced at the active sites or the edges of MoS_2_ during the reaction in situ functionalization, resulting in increased catalytic and electronic properties [119]. In situ synthesized NPs are smaller in size and are more uniformly dispersed in the matrix, resulting in better mechanical properties [120,121]. Improved spatial distributions of NPs on the surface of the MoS_2_ nanosheet not only increase the synergistic effect and active surface area but also have the potential to control the charge carrier density [88,122]. As a result of enhanced electrical conductivity and electron mobility, as well as a large active surface area, biological molecules can be effectively immobilized on MoS_2_ for the detection of target analytes [123].

The benefit of the in situ approach is the prospect of using a range of chemical synthesis approaches, including deposition methods, hydrothermal/solvothermal techniques, sol–gel synthesis, etc. [111]. Unlike ex situ functionalization, it is possible to avoid the use of surfactants and capping agents during the in situ synthesis of NPs. The drawback of this method is that the unreacted precursors or by-products of the in situ reactions might influence the properties of the final material [124,125].

**Figure 3:**
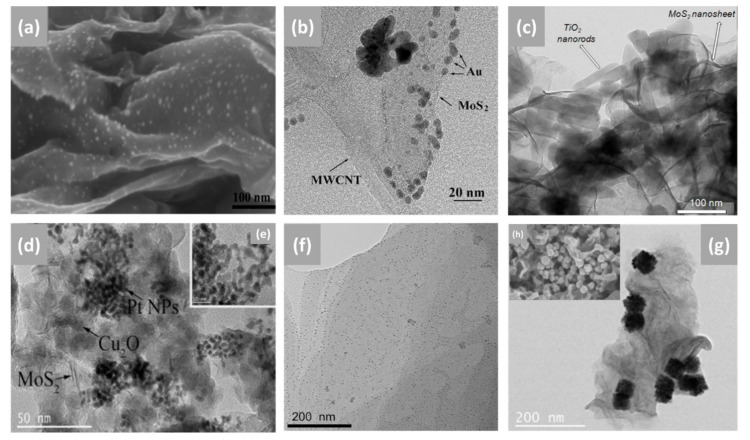
SEM image of (**a**) MoS_2_-Au [13], TEM images of (**b**) MoS_2_-MWCNT/Au [83], (**c**) MoS_2_-TiO_2_ [106], (**d**) MoS_2_-Cu_2_O/Pt, ((**e**) insert Pt NPs) [70], (**f**) N/F/MoS_2_-Ag [88], and (**g**) MoS_2_-PtPd ((**h**) insert SEM of MoS_2_-PtPd) [96], produced by in situ functionalization. Figures reproduced with permission from Ref. [13] copyright © 2016 American Chemical Society, Refs. [70,83] copyright © 2017 Elsevier B.V., Ref. [106] copyright © 2017 Published by Elsevier Ltd., ref. [88] copyright © 2018 Elsevier B.V., Ref. [96] copyright © 2019 Elsevier B.V.

Among various articles published on the in situ preparation of numerous different nanocomposites in the application of electrochemical sensors, electrodeposition is among the most commonly used method for surface modification. Electrodeposition uses electrical currents to reduce metallic ions from the electrolyte solution of a specific material and coat the material as a thin film onto a conductive substrate surface (electrode) [126,127,128]. Overall, the electrodepositing process may either be: (i) an anodic process in which a metal anode in the solution is electrochemically oxidized, reacts together, and then deposits on the anode; or (ii) a cathodic process in which components (ions, clusters or NPs) are deposited from solution precursors onto the cathode [128]. This method has already been used for the deposition of the NPs on the 2D MoS_2_ surface [83,84,85,86]. The most significant feature of the process is that the morphology and film thickness of NPs can be conveniently tuned by adjusting the experimental parameters, for instance, the film thickness of NPs can be regulated by the electrodeposition time, and the rate of deposition can be influenced by the current changing over time [128]. Moreover, Bera et al. [129] mentioned that the ultimate size distribution of the electrodeposits is strongly dependent on the kinetics of the nucleation and growth. Furthermore, during the process of electrodeposition, the nucleation of NPs on the electrode substrate is determined by the structure of the substrate, specific free surface energy, adhesion energy, and lattice orientation of the electrode surface [129]. Additionally, this single-step method has some benefits, such as low-cost equipment, a relatively low processing temperature, short deposition time, and reduced material waste [130,131]. It has been reported that electrodeposition is a better method compared to drop casting when it comes to electrode modification. The biosensor prepared by the electrodeposition method has a fast response time, and a 50-fold lower detection limit compared to the biosensor prepared by the drop casting method. Other attributes include high reproducibility, long-time storage stability, and adequate selectivity, suggesting that the electrodeposition of the nanocomposite provides a promising route to novel types of highly sensitive and stable electrochemical biosensors [132]. Su et al. [13], fabricated an electrochemical aptasensor based on Au NPs decorated on MoS_2_ nanosheets. The Au-MoS_2_ nanocomposite was formed by the electrodeposition of Au NPs on MoS_2_ nanosheets. After an electrodeposition cycle, the scanning electron microscope (SEM) image (Figure 3a) showed that Au NPs with an average diameter of 10 nm were distributed homogeneously over the surface of MoS_2_ nanosheets, indicating that the MoS_2_ was successfully functionalized. Zhang et al. [83] developed an electrochemical sensor for the detection of nitrite by electrochemical deposition of Au NPs onto the surface of MoS_2_-MWCNT nanocomposite. As shown by the TEM image in Figure 3b, Au NPs with sizes ranging from 3 to 5 nm were successfully grown on the MoS_2_-MWCNT nanocomposite. However, the Au NPs were not evenly distributed and showed some agglomeration.

Other popular methods for the functionalization of 2D MoS_2_ nanosheets with NSs include hydrothermal/solvothermal and chemical reduction. Hydrothermal/solvothermal syntheses are described as conducting chemical reactions in solvents placed in sealed vessels in which solvent temperatures can be brought up to their critical points by heating concurrently with autogenous pressures; the method is known as hydrothermal when water is used as a solvent [133,134]. Syntheses of materials by hydrothermal/solvothermal methods involve crystallization directly from solutions that typically require two steps: crystal nucleation and subsequent growth. The resulting nanomaterials may be produced with required particle sizes and morphologies by regulating processing variables, such as temperature, pH, reactant concentrations, and additives [53]. Figure 3c exhibits reported TEM results of MoS_2_ nanosheets decorated with titanium dioxide (TiO_2_) nanorods by the hydrothermal process. The MoS_2_-TiO_2_ nanocomposite image showed that the uniform cylindrical TiO_2_ nanorods with an average diameter of ~20 nm and length of ~180 nm were incorporated and partially dispersed into the MoS_2_ nanosheets [106]. In another study, MoS_2_ nanosheets were functionalized by the solvothermal reaction and post-immobilization process. Firstly, MoS_2_ nanosheets were coated with 15 nm Copper(I) oxide (Cu_2_O) NPs via solvothermal synthesis, the synergistic interaction between MoS_2_ and Cu_2_O facilitated the formation of a homogeneous structure of a small particle size, as shown in Figure 3d. Formation of MoS_2_-Cu_2_O/Pt nanocomposite was by post immobilization of a platinum (Pt) NP solution under ultrasonic conditions. From the reported TEM results (Figure 3d,e), Pt NPs with an average diameter of 3 nm were partially dispersed on MoS_2_-Cu_2_O nanocomposite [70].

Some researchers opt for chemical reduction syntheses for the functionalization of MoS_2_ nanosheets. For instance, Figure 3f,g exhibit the reported TEM results of a 2D MoS_2_ nanosheet decorated with silver (Ag) NPs and PtPd bimetallic nanocubes (PtPd NCs), respectively. The insert image (Figure 3h) is the SEM image of MoS_2_-PtPd. Ag NPs (diameter 3 nm) were homogeneously dispersed on the surface of the MoS_2_ nanosheet and there were none of the Ag NPs outside the nanosheet, which could be due to enhanced MoS_2_ surface activity resulting from binary co-doping of nitrogen fluorine elements [64]. On the other hand, PtPd NCs with an average side length of about 50 nm were partially distributed on MoS_2_ nanosheets, and the surface of PtPd NCs was not smooth, as it consisted of smaller NCs instead of cubic nanosheets [96]. The chemical reduction method is highly favored because it is simple, cost-effective, and has the advantage of producing NPs without aggregation. Moreover, it can achieve better particle size and distribution control by simply optimizing the experimental parameters, such as the molar ratio of the capping agent with the precursor salt and the ratio of the reducing agent with the precursor salt [135]. This method includes the reduction of metal salt in a suitable medium in the presence of a surfactant/stabilizer and a reducing agent [136,137].

While researchers aim for the functionalization of 2D MoS_2_ nanosheets to broaden their prospective applications, it is important to note that control of catalytic, electronic, and optical properties of these nanomaterials during production is crucial for device applications, irrespective of the method of synthesis and functionalization.

## 4. Properties

The 2D nanomaterials are known to have special properties, which are distinct from their 3D bulk counterparts. The 2D MoS_2_ nanosheets with completely tunable properties can be synthesized successfully with various types of methods as discussed above. Due to changes in properties with a decrease in the number of layers, 2D MoS_2_ nanosheets possesses significantly distinctive electrical, optical, and chemical properties, which are known to play important roles in the fabrication of sensors. The exceptionally large surface areas of 2D MoS_2_ nanosheets offer robust surface functionalization and provide new possibilities for functional devices based on 2D materials. MoS_2_ has been reported to simply form nanocomposites with metal and metal oxide NSs [69,82], carbon nanomaterials [137,138], conductive polymers [139,140,141], and other 2D nanomaterials [142,143], which could be used as catalytic materials to significantly increase the electrochemical performance of sensors based on MoS_2_-nanocomposites. The 2D MoS_2_/metal-NS composites have the inherent properties of pure metal NSs and MoS_2_ nanosheets due to their synergistic effects, which makes the 2D MoS_2_/metal-NS composites exhibit outstanding electrochemical properties and improved sensitivity [13]. Through studying Zhang’s review paper [8], one can find comprehensive insights into MoS_2_ nanosheets used as building blocks or supports for the preparation of MoS_2_ nanosheet-based composites with other materials.

### 4.1. Electrical Properties

The bulk MoS_2_ is considered to have low electrical conductivity and high electrical resistivity mainly due to its intrinsic structure, where single sheets of MoS_2_ are stacked together and bonded by week van der Waals interactions between adjacent single sheets. However, 2D MoS_2_ has a unique morphology and electrical properties with efficient transmission of electrons on account of the direct energy band structure. Thus, an understanding of the electronic structure of 2D MoS_2_ layers is of great importance. For instance, changes in the interlayer coupling, degree of quantum confinement, and symmetry elements result in significant differences in 2D MoS_2_ electronic structure relative to bulk MoS_2_ [9,144,145,146]. The electronic structure of the single-layer and few-layer MoS_2_ and the resulting special optical properties arise from the configurations of the d-electron orbitals that comprise the MoS_2_ conductive and valence bands [147,148]. The outer d-electron interactions give rise to a band gap of ~1 eV between the occupied and unoccupied “d” states of single-layer MoS_2_, which make them different from other sp-bonded semiconductor nanostructures [9,149]. TMDs have diverse electronic properties that emerge from the gradual filling of non-bonding d bands from group 4 to group 10 transition metals. When the orbitals are partially filled, as in the case of 2H-NbSe_2_ and 1T-ReS_2_, TMDs exhibit metallic conductivity. When the orbitals are fully occupied, such as in 1T-HfS_2_, 2H-MoS_2_, and 1T-PtS_2_, the materials are semiconductors [149]. The MoS_2_ band structures can be calculated from the first principles of the density functional theory (DFT) in order to obtain more information regarding their electronic structure.

**Figure 4:**
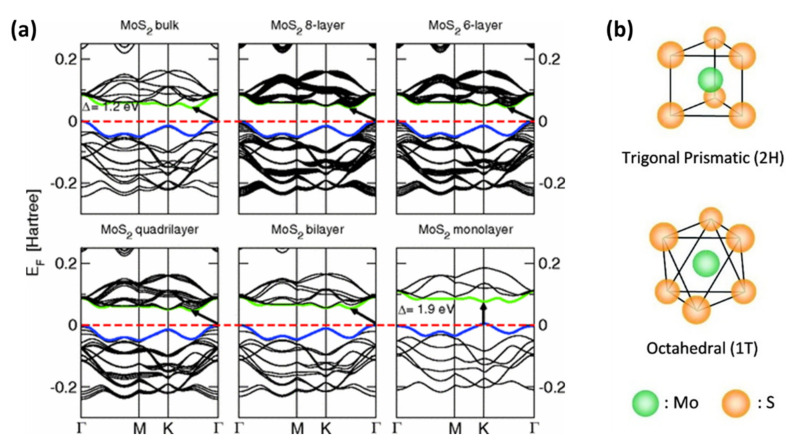
(**a**) The band structures of MoS_2_ from bulk to monolayer MoS_2_ calculated at the DFT-PBE level. The horizontal dashed lines indicate the Fermi level. The arrows indicate the smallest value of the band gap (direct or indirect) for a given system. The green and blue lines represent the conduction and valence band edges, respectively [146]. (**b**) Structure of 2H- and 1T-MoS_2_ [46]. Figures reproduced with permission from Ref. [146] copyright © 2011 American Physical Society, and Ref. [46] copyright © 2011, American Chemical Society.

Kadantsev et al. [145] investigated the electronic structure of a single-layer MoS_2_ with all electron first-principle calculations based on DFT and variational treatments of spin-orbital coupling. Another detailed study was conducted on the effect of quantum confinement on the electronic structure of single-layer and few-layer MoS_2_. The reported results indicated that the band gap of the bulk MoS_2_ stemmed from the transition from the top of the valence band situated at Γ to the bottom of the conduction band halfway between the Γ and K high symmetry points, and the optical direct band gap of the single-layer MoS_2_ was situated at K point [146]. The conductive band states at the K point consist mainly of highly localized d orbitals at the Mo atom sites located in the middle of the S-Mo-S unit cell and they have minimal interlayer coupling [148]. However, the states near the Γ point are attributed to a linear combination of d orbitals on Mo atoms and antibonding pz orbitals on S atoms and they have strong interlayer coupling with energies that depend sensitively upon the thickness of the layers [148]. Figure 4a illustrates the band structures obtained for bulk to 2D MoS_2_. The trend observed is that the direct excitonic transition energy at the Brillouin zone K point hardly changes with the thickness of the layers; however, decreasing the number of layers leads to a gradual shift in the indirect band gap to a high direct band gap in mono-layers that the material turns into a 2D semiconductor [146,148]. This shift from the bulk indirect band gap to a single-layer direct bandgap results from the effects of quantum confinement [144,146].

In addition to studying the electronic structures of the bulk MoS_2_ and the 2D MoS_2_, the fundamental experiments and theoretical studies have suggested that other variables may affect the material’s electrical properties, such as preparation methods [44,150,151,152], functionalization or doping [122,153,154,155,156], and environmental conditions [157]. For instance, many CVD-based methods for the synthesis of 2D MoS_2_ have been developed, and changing experimental conditions, such as varying degrees of MoO_3_ sulfurization during formation, can affect the material’s optical and electrical properties [150]. As reported, the Li intercalation of MoS_2_ leads to the structural transformation of MoS_2_ from the 2H phase to the 1T phase (Figure 4b) triggered by the charge transfer from Li to MoS_2_ [149], as well as a transition from semiconductor to metallic 2D MoS_2_ nanosheets [43,44,46]. The intercalation process usually produces negatively charged nanosheets with different properties from their neutral counterparts. The antibonding d-orbitals of the transition metals are filled leading to different electronic properties, such as the change from semiconducting nanosheets to metallic [158]. Ambrosi et al. [44] studied the influence of Li intercalation on the electrochemical properties of exfoliated MoS_2_ nanosheets. The electrochemical measurements were conducted with a MoS_2_-modified carbon electrode (vs. Ag/AgCl reference electrode) using the cyclic voltammetry (CV) method over a potential range of −1.6–1.4 V. From the results obtained, n/tert-butyllithium (Bu-Li)-exfoliated MoS_2_ exhibited a large current density with oxidation and reduction peak intensities almost three times greater than the bulk material. These intrinsic electrochemical properties of exfoliated MoS_2_ have significant implications as they could create new opportunities in the field of electrochemical sensing and biosensing where the anodic and cathodic signals from MoS_2_ can be used as identification and quantification tags [44]. The major influence on 2D MoS_2_’s electrical properties could also be due to considerable environmental effects, and such effects could greatly restrict the exploration of 2D MoS_2_’s inherent properties. Khan et al. [157] studied the electrical and photoelectrical properties of MoS_2_-based field effect transistors (FETs) in the dark and in the presence of deep ultraviolet (DUV) light under various environmental conditions (vacuum, N_2_ gas, air, and O_2_ gas). The results obtained showed the difference in device performance. In the dark, the environmental gases did not modify the I_on_/I_off_ of the device; however, the environmental gases changed the characteristics of the device under DUV illumination. Further studies were also carried out to evaluate the influence of temperature and pressure on the electrical conductivity of 2H-MoS_2_ [159,160].

While many other relevant studies are being conducted on MoS_2_ electrical properties, there are very few detailed studies focused on the effects of doping or functionalization on optical and electrical properties of bulk and 2D MoS_2_. Doping or functionalization are considered some of the most common ways to alter the band structures of semiconductor materials [161]. The large surface areas of 2D materials offer effective surface functionalization and create new possibilities for functional devices. In particular, MoS_2_ functionalized with metal-based NPs was found to have additional benefits in potential applications, such as electrochemical sensors [69,108]. 

**Figure 5:**
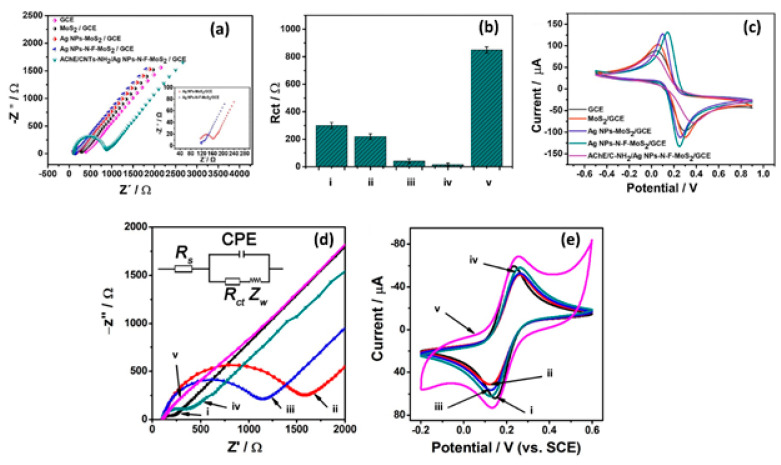
(**a**) EIS response of bare and modified electrodes with a frequency range from 0.01 to 100 kHz, with an amplitude of 5 mV. (**b**) The histogram for R_CT_ of (i) bare/GCE, (ii) MoS_2_/GCE, (iii) Ag NPs-MoS_2_/GCE, (iv) Ag NPs-N-F-MoS_2_/GCE, (iii) AChE/CNTs-NH2/Ag NPs-N-F-MoS_2_/GCE. Error bars are coefficients of variation across three repetitive experiments. (**c**) CV response of bare and modified electrodes at a scan rate of 100 mV s^−1^ [88], (**d**) EIS and (**e**) CV responses of (i) bare/GCE, (ii) MoS_2_/GCE, (iii) Au-MoS_2_/GCE, (iv) Pd-MoS_2_/GCE, and (v) Au-Pd-MoS_2_/GCE [89]. Figures reproduced with permission from Ref. [88] copyright © 2018 Elsevier B.V, and Ref. [89] copyright © 2016 Elsevier B.V.

When it comes to electrochemical sensors, researchers typically use electrochemical techniques, such as CV, to study electron transfer kinetics, and electrochemical impedance spectroscopy (EIS) to study the charge transfer resistance (R_ct_) of the material on the electrode surface in K_3_[Fe(CN)_6_]/K_4_[Fe(CN)_6_] electrolyte. Song et al. [88] utilized nitrogen fluorine pre-doped 2D MoS_2_ nanosheets decorated with Ag NPs (Ag NPs-N-F-MoS_2_). Upon characterization with EIS, he found a decrease in R_ct_ of the AgNPs-MoS_2_ (40 Ω)-modified glassy carbon electrode (GCE) as compared to the non-functionalized MoS_2_ (220 Ω) (Figure 5a,b).

These findings indicate that the synergistic effect between MoS_2_ nanosheets and Ag NPs has induced enhancement of the signal and increased electrical conductivity, suggesting that MoS_2_ electrical properties can be effectively tuned by incorporating Ag NPs with MoS_2_ [88]. Similar results were observed with CV characterization (Figure 5c). The CV responses of modified electrodes showed typical K_3_[Fe(CN)_6_]/K_4_[Fe(CN)_6_] redox peaks with narrow peak-to-peak potential separations. The fast electron transfer rate was obtained at the AgNPs-MoS_2_-modified electrode, indicating a good electrical conductivity of the AgNPs-MoS_2_ nanocomposite. The most intriguing finding was an increased electron transfer rate and a low R_ct_ (15 Ω) response in Ag NPs-N-F-MoS_2_. This suggests that non-metal dopants affect the electrical characteristics of MoS_2_. Theoretical calculations in the study revealed that nitrogen fluorine pre-doped 2D MoS_2_ nanosheets had smaller bang gaps (0.8 eV) than pristine MoS_2_ nanosheets (1 eV). This proved that by doping MoS_2_ nanosheets with non-metal dopants, excellent intrinsic conductivity could be achieved. Furthermore, doping allowed for good interfacial bonding and monodispersity of Ag NPs on N-F-MoS_2_, increasing the surface area and preventing side reactions. As a result, excellent conductivity, exceptional electron mobility, and fast mass transport were achieved.

In another study, MoS_2_ nanosheets functionalized with Au-Pd bimetallic NPs were applied and tested for the interface properties of modified electrodes using EIS (Figure 5d). The reported results showed that electrodes modified with MoS_2_ had a high R_ct_ of 1373 Ω due to poor conductivity and negative MoS_2_ nanosheets charges. After post-electrode modification with Au-Pd/MoS_2_ nanocomposites, R_ct_ was significantly reduced due to the excellent conductivity of bimetallic Au-Pd NPs. The CV (Figure 5e) results matched those of the EIS. MoS_2_ nanosheets exhibited low current responses compared to Au-Pd/MoS_2_ nanocomposites with significantly higher current responses [89]. It was reported that the NP coverage on the MoS_2_ nanosheet surface has the ability to control the charge carrier density, which could have a major effect on MoS_2_-based sensor fabrication and device optimization [153]. Chamlagain et al. [153] studied the control of interfacial charge transfer and tailoring of the electronic charge transport properties of the MoS_2_ FET based on the functionalization of MoS_2_ by varying the coverage of Au NPs on the MoS_2_ surface. The results showed increasing Au NPs coverage; the threshold voltage of the MoS_2_ FET showed a continuous positive change from 4 to 41 V, resulting in a tunable charge transfer of 3.5 × 10^11^/cm^2^ to 4 × 10^12^/cm^2^. The carrier mobility also varied from 52.5 to 2.8 cm^2^/VS with Au NP coverage. Another study indicated that doping induces large variations in the electrical properties of 2D TMDs [122]. The electrical properties (e.g., resistivity) would largely depend on the numbers and positions of the dopant atoms in 2D TMDs, which is very challenging due to the diffusion laws and the statistical complexity of dopant distribution [122]. However, there is still a lack of intensive research on the effect of NP coverage on the MoS_2_ surface and the role of dopant atoms on electrical properties in 2D TMDs.

### 4.2. Optical Properties 

**Figure 6:**
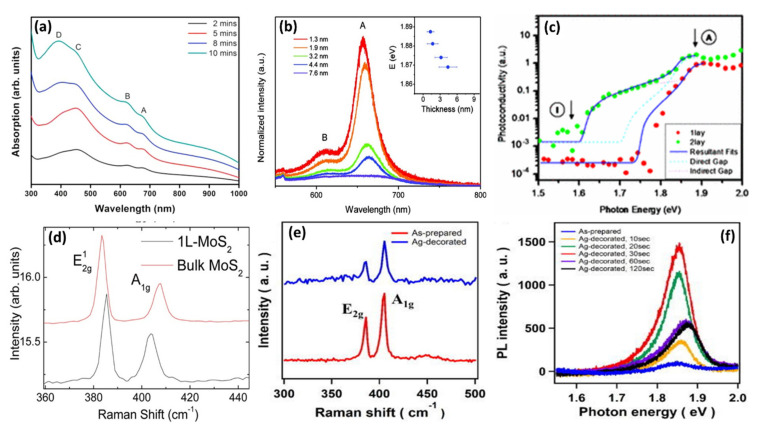
(**a**) UV–visible spectroscopy spectra of MoS_2_ layers [168], (**b**) PL spectra of MoS_2_ thin films with thicknesses ranging from 1.3 to 7.6 nm [46], (**c**) photoconductivity spectra for mono (red dots) and bilayer (green dots) MoS_2_ [144]. (**d**) Raman spectra for (top) bulk MoS_2_, and (bottom) 1L-MoS_2_ [167], (**e**) Raman spectra of MoS_2_ before (red) and after (blue) Ag NP decoration [161], (**f**) PL spectra of MoS_2_ decorated with Ag NPs [161]. Figures reproduced with permission from Ref. [168] copyright © 2017 Elsevier, Ltd., Refs. [46,144] copyright © 2011, American Chemical Society, Ref. [167] copyright 2019–2020 Aptara, Inc. and Ref. [161] rights managed by AIP Publishing, Woodbury, NY, USA.

MoS_2_ nanomaterials have since surfaced as potential materials for several applications in optical devices due to their impressive optical properties. The direct 1.9 eV optical gap in 2D MoS_2_ results in an electron–hole pair excitation that could be used in LEDs, photodetectors, and other photonic devices [147,162]. The stability of MoS_2_ towards photocorrosion is beneficial in photochemical reactions and the tunable band gap indicates that the material can harvest light over a wide range, from ultraviolet to infrared wavelengths [149]. The stability of MoS_2_ towards photocorrosion is considered to be due to its antibonding characteristics. According to the extensive dichalcogenide band structure calculations, the relevant Γ^−^_4_ state at the top of the valence band is not a non-bonding metal d state but rather an antibonding state between metal $d_{z}^{2}$ and non-metal $p_{z}\;$ orbitals [162]. There are several factors that can directly influence MoS_2_’s optical properties, such as the electronic band structures, as described previously, quantum confinement effects, and functionalization or doping [6,149,155,162]. The quantum confinement triggers a distinct change in electronic or optical properties in nanomaterials [163]. The quantum confinement effect has been reported to induce a strong blue shift as large as 4 eV in MoS_2_ optical absorption features when the lateral dimensions of the MoS_2_ nanostructures are reduced to nanometers [147,162,164]. These unique optical properties come from the d-electron orbitals that dominate the valence and conduction bands [146]. Extensive experimental studies have been carried out using spectroscopic techniques, such as optical absorption, photoluminescence (PL), photoconductivity, spectroscopic ellipsometry (SE), and electroluminescence to better understand the optical properties of bulk and single-layer MoS_2_ [46,144,165,166,167]. The Raman signal is also used to confirm the atomic structural arrangement of MoS_2_ and to provide potential explanations of layer-dependent optical properties [15,54,148,152]. The increase in bandgap energy due to the change in indirect to direct bandgap induces changes in absorption spectra. Figure 6a shows the optical absorption spectra of MoS_2_ deposited on a SiO_2_/Si substrate with varying atomic layer thicknesses using a chemical bath deposition (CBD) technique. The thicknesses of the atomic layers increased as the deposition time increased from 2 to 10 min. The number of layers ranged from 2, 3, 4, to a few layers, with time varying between 2, 5, 8, and 10 min, respectively. The optical absorption spectra exhibited two distinctive absorbance peaks assigned to exciton bands A and B between 600 and 700 nm [46,167,168]. These excitonic peaks were considered to result from direct gap transitions between the maxima of split valence bands and the minimum of the conductions all positioned at the K point of the Brillouin zone [144,148]. However, only a slight change in absorption peaks from the direct excitonic states was observed with change in the number of MoS_2_ layers [144,148]. The A and B exciton peaks in the single-layer MoS_2_ were found to shift upwards in energy relative to those of the bulk counterpart and had smaller separation due to reduced interactions between the layers [169]. The C and D absorption peaks may be attributed to the direct transition from the deep valence band to the conduction band [168]. In PL studies, it was discovered that the photoluminescence of MoS_2_ increased with a decreasing number of layers/layer thickness, and that the luminescence from a single-layer MoS_2_ is more enhanced, whereas in bulk material it is absent [6,144,148]. Figure 6b shows the PL spectra of MoS_2_ thin layers with thicknesses ranging from 1.3 to 7.6 nm (single layer ~0.65 nm thickness, (6.5 Å)). The energy of the A exciton peak as a function of average layer thickness is shown in the inset. The PL spectrum of MoS_2_ exhibits one enhanced peak and one small peak at around (A) 660 nm (1.9 eV) and (B) 610 nm (1.77 eV), respectively. These emission peaks are in perfect alignment with the energy of the excitons A and B [46]. The enhanced peak relates to the band gap of the materials, and the small peak may be due to the valence band splitting triggered by strong spin–orbit coupling, which corresponds to the direct excitonic transition at the K point of the Brillouin zone of MoS_2_ [168]. The photoluminescence behavior of this material suggests that the luminescence quantum efficiency is much higher in single-layer/few-layer MoS_2_ than in multilayer MoS_2_ [148]. 

The photoconductivity of these materials also show characteristics related to the band structure. According to the photoconductivity results (Figure 6c) by Mak et al. [144], the conductivity of MoS_2_ increases steadily with respect to photon energy towards the direct band gap, as anticipated for an indirect band gap material. These characteristics indicate that bilayer MoS_2_ is an indirect band gap semiconductor, whereas single-layer MoS_2_ is a direct band gap material [151]. Splendiani et al. [148] observed PL and Raman signal dependence on MoS_2_‘s number of layers. The Raman features, i.e., the frequency, intensity, and width of the peaks, are highly influenced by the number of layers. Raman spectrum in Figure 6d exhibits two main peaks at 385 cm^−1^ and 404 cm^−1^ corresponding to the in-plane ($E_{2g}^{1}$) vibrational mode of S atoms and out-of-plane ($A_{1g}$) vibrational mode of Mo and S atoms, respectively [167,168]. The frequency of the $A_{1g}$ vibrational mode increases as the number of layers of MoS_2_ increases to bulk, while the frequency of the $E_{2g}^{1}$ vibrational mode decreases [165,169]. Moreover, a blue shift is observed with the $A_{1g}$ vibrational mode, while the $E_{2g}^{1}$ vibrational mode displays a red shift. The shift in frequencies was attributed to long-range Coulombic interlayer interactions and the influence of stacking-induced structural changes [170]. A review by Ye et al. can be studied for more knowledge on optical properties of 2D MoS_2_ [171]. Kim et al. [161] studied the optical properties of MoS_2_ functionalized with Ag NPs. The reported Raman results (Figure 6e) indicated that the structure of MoS_2_ was not affected by Ag NPs because the Raman shift is almost identical before and after decoration with Ag NPs. However a slight upshift of the $A_{1g}$ emission peak was observed, indicating a p-type doping effect. The PL results (Figure 6f) showed that Ag NPs enhances the excitonic emission of MoS_2_; however, upon an increase in irradiation time from 10 to 120 s, PL intensity decreased due to the light blockage by overloading of Ag NPs on the surface of MoS_2_.

**Figure 7:**
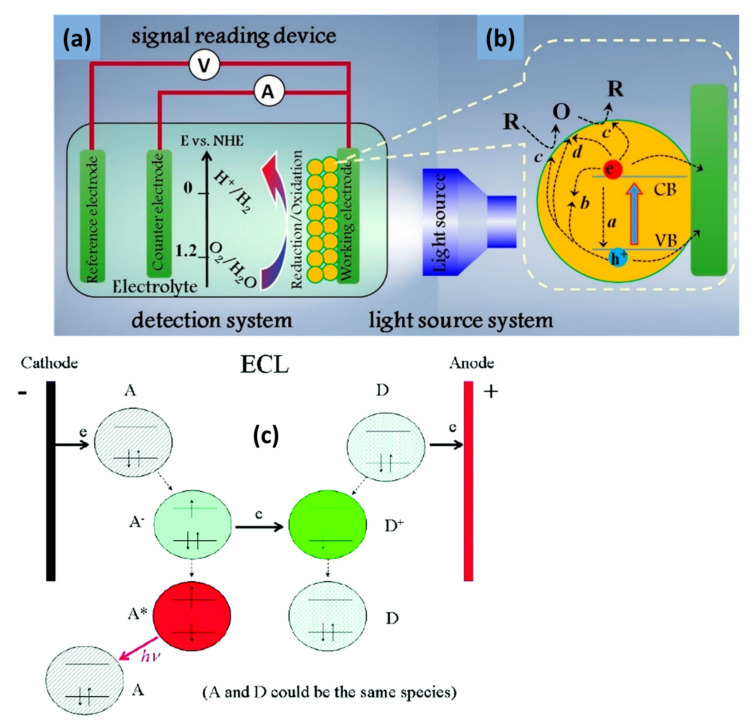
(**a**) Schematic representation of PEC sensing with a traditional three-electrode system, (**b**) the photocurrent generation mechanism, [173], and (**c**) schematic representation showing the general principles of ECL [184]. Figure reproduced with permission from Ref. [173] copyright © 2020 American Chemical Society, Ref. [184] copyright © 2007 Elsevier B.V.

These optical properties play vital roles in photoelectrochemical (PEC) and electrochemiluminescence (ECL) sensors for the detection of various biological molecules. The optical absorption properties of PEC sensors based on 2D-MoS_2_/metal-NSs composites are typically studied by using the photocurrents generated when exposing the modified electrodes to intermittent visible light. PEC’s general mechanism is based on the photo-to-current conversion that occurs from the excitation of the electrons and the resulting charge transfer of a material after photons are absorbed. As light excites photoelectrochemically active materials with enough energy to produce electron–hole pairs, charge separation and charge transfer will occur to the electrode and solution interface with oxidation-reduction reaction, generating photocurrent or photovoltage, which is the reverse of ECL processes [172,173,174,175]. A typical sensing system comprises of three important components: excitation light source system, the detection system (electrolyte, metal electrode with electrical catalytic activity, and the photoactive material modified working electrode), and the signal reading instruments, as shown in Figure 7a,b [173,175]. Once the electron−hole pair is formed, the discharge of the conduction band electrons into the electrode, with the concurrent transfer of electrons from the electron donor in the solution, results in an anodic photocurrent. On the other hand, the transfer of the conduction band electrons to the electron acceptor in the solution, followed by the supply of electrons from the electrode to neutralize the valence band holes, result in a cathodic photocurrent (Figure 7a,b) [172]. The 2D MoS_2_ has been used as a photocatalyst in PEC sensors and the material has proven to have great potential in PEC applications [176]. Even so, studies have shown that another way to improve photocurrent is to integrate metal-based NSs as they can enhance the conductivity of semiconductor materials and promote the separation of photogenerated electron–hole pairs [177]. For example, Au NPs have proven to enhance the photocurrent response of 2D MoS_2_ due to their excellent conductivity, surface plasmon resonance effect, energy transfer effect, and superior binding capabilities [80,178]. TiO_2_ NPs have also emerged as materials of interest to enhance the photocurrent response of 2D MoS_2_-based PEC sensors. Studies suggested that the matched energy band of the MoS_2_-TiO_2_ nanocomposite favors the charge transfer and restricts photogenerated electron and hole recombinations between MoS_2_ and TiO_2_ under visible light radiation, leading to the enhanced photocatalytic activity [106,179]. In general, ideal photoactive materials have high visible-light absorption capacities and exhibit fast charge transfers in order to separate the electron/hole pairs and suppress charge recombination. 

In the ECL process, a light is emitted when the electrochemical species on the electrode surface form excited states from undergoing a high energy (exergonic) electron transfer reaction [180,181,182]. The ECL process is based on the electroluminescence method for detecting the light intensity while the PEC analysis detects the photocurrent. ECL has distinct advantages over other light emission techniques, such as PL and standard chemiluminescence (CL). In particular, ECL has excellent temporal and spatial control on light emission compared to CL. In addition, the absence of excitation light in ECL offers near-zero background noise, whereas PL suffers from non-selective photoexcitation-induced background noise [181,182]. ECL reaction pathways are generally divided into two categories: annihilation ECL and co-reactant ECL. One can study the reviews by Rizwan et al. [183] and Miao [184] for detailed discussions of these two pathways. Despite the different ECL pathways, there are usually four steps involved in most ECL processes, which include redox reactions at the surface of the electrodes, homogeneous chemical reactions, excited state species formation, and light emission [185]. In the general principles of ECL (Figure 7c), species A accepts one electron from the cathode to form A^•−^, and species D loses one electron at the anode to form D^•+^. When A^•−^ and D^•+^ diffuse away from the electrodes and come together, A^•−^ transfers one electron to D^•+^ to produce a neutral species D and the excited state A*. A* immediately emits light (hv) and returns to its ground state [183,184]. The direct (or indirect) application of photoactive nanomaterials to ECL sensors has become popular. The incorporation of nanocomposites into ECL sensors has been shown to influence the sensitivity, stability, detection range, and detection limit of the sensing platform [183]. The 2D MoS_2_ nanosheets have the advantages of increasing conductivity, providing a high surface area, and improving the photocatalytic performance of ECL sensors [81,186,187]. However, only a few studies on ECL sensors based on 2D-MoS_2_/metal-NSs composites have been reported. The application and effect of metal-based NSs on 2D MoS_2_ in PEC and ECL sensors is discussed extensively in Section 5.

### 4.3. Catalytic Properties 

MoS_2_ nanomaterials have been of interest for various applications. In addition to photoelectrochemical [80] and photoluminescence [81] properties, MoS_2_ became particularly notable for its catalytic properties toward the hydrogen evolution reaction (HER) [33]. Therefore, a significant amount of work is aimed at improving the 2D MoS_2_’s catalytic properties underpinned by the possibility of replacing Pt as a catalyst. The 2D MoS_2_ is a promising non-precious material as opposed to conventional noble metal catalysts because of its high chemical stability, cost effectiveness, and good catalytic efficiency. The catalytic ability of inherent 2D MoS_2_ may not be as high as that of noble metals but shows good resistance to poisoning [188]. The advantages of 2D MoS_2_ nanosheets are the abundant exposed edges, which have been identified as the bases of their catalytic activity [60,61]. While bulk MoS_2_ is relatively inert, the catalytic activity of 2D MoS_2_ is localized to rare surface sites [189]. The identification of active sites is important for evaluating a catalyst’s performance; hence, numerous attempts have been made to synthesize MoS_2_ nanomaterials with large edge sites and improved catalytic properties. For instance, MoS_2_ thin film quantum dots (QD) have been used as catalysts for HER. Due to possible defect-rich characteristics and having more active edge sites, the MoS_2_ QD exhibited the superior performance as an electrocatalyst [168]. It has been reported that another way of regulating MoS_2_’s catalytic properties is through shape and size control [190]. The fundamental size-dependent properties of nanomaterials arise from changes in the electronic structures that result from the spatial confinements of electrons within very small nanoclusters. When the MoS_2_ sheet is reduced in size, it gives rise to nanosheets with low-coordination step-edges, kinks, and corner atoms that induce additional local chemical effects [149,190]. The quantum size effects also induce shifts in the valence band and the oxidation potentials, permitting catalytic activities not possible with the bulk band structure [149]. Through a series of catalyzed reaction steps on low-coordinated sites on MoS_2_ nanoclusters, Tuxen et al. [190] studied how the adsorption properties of MoS_2_ nanoclusters toward the hydrodesulfurization (HDS) refractory dibenzothiophene (DBT) differ intensely with small changes in the sizes of nanoclusters. The results showed that bigger MoS_2_ nanoclusters with more than 6 Mo atoms on the edge of the cluster displayed a preference for S vacancy formation on the edges, but those edge vacancies did not appear to have any affinity for binding DBT because of their steric hindrances. However, smaller MoS_2_ nanoclusters, with less than the threshold value of 6 Mo atoms, were found to form vacancies mainly on corner sites; this was seen as a favorable situation because the unconstrained access to Mo at such corner S vacancy sites enabled strong adsorption of DBT molecules.

Chia et al. [191] studied catalytic properties of 1T metallic MoS_2_ materials via electrochemical methods toward HER. The study showed that the metallic 1T phase of MoS_2_, produced by chemical exfoliation through lithium intercalation, enhanced the catalytic activity over the semiconducting 2H phase due to the better conductivity properties that induced the charge transfer kinetics. The ball milling method has been reported to produce MoS_2_ nanostructures with enhanced catalytic properties towards HER [192,193]. Since the catalytic activity of solid catalysts is facilitated by active sites, which are often described as surface defects, ball milling has been widely used to increase catalytic activity by producing more lattice defects and dislocations [192]. It was discovered that the produced MoS_2_ nanostructures have distorted structures with an abundance of exposed edge sites, showing outstanding electrochemical activity in the HER with a high current density [192,193]. In addition to the methods discussed above to improve the catalytic properties of MoS_2_, other approaches involve functionalization of MoS_2_ nanosheets with metal-based NSs [60,115] or edge-site substitutions with transition metals to form bimetallic catalytic sites [194]. Functionalization of MoS_2_ nanosheets with metal-based NSs is the common approach to improve electrocatalytic properties of an electrochemical sensor. These 2D MoS_2_/metal-NS composites have the essential properties of MoS_2_ nanosheets and pure metal NPs due to their synergistic effects, resulting in excellent electrocatalytic properties of 2D MoS_2_/metal-NS composites. For instance, the MoS_2_-Au nanocomposite was reported to have an improved electrocatalytic response towards the detection of glucose. The MoS_2_-Au nanocomposite also provided additional electrochemical properties, such as high faradic-to-capacitive current ratios, high current density and electron mobility, and faster mass transport [69]. The MoS_2_-Cu_2_O-Pt nanocomposite was reported to have several features of optimized interfacial contacts compared to single-metal oxide NPs and MoS_2_ nanosheets. The combination of Cu_2_O NPs and MoS_2_ nanosheets significantly improved the electronic transmission capability, specific surface area, catalytic performance, and dispersibility of the MoS_2_-Cu_2_O nanocomposite [70]. In another study, the deposition of Cu NPs on the high specific surface area MoS_2_ nanosheets exhibited increased electrocatalytic activity toward oxidation of biological molecules due to the synergic effect between Cu NPs and MoS_2_ nanosheets [108]. Therefore, MoS_2_ and other TMDs nanomaterials are considered to represent a new range of highly electroactive materials. Their nanocomposites with other forms of catalytic nanostructures, such as metals and metal oxide NSs, could further progress to improve performance in electrochemical sensing. The current electrochemical sensors based on 2D-MoS_2_/metal-NSs composites can be categorized into several types, including, electrochemical biosensor, PEC sensor, ECL sensor, and electrochemical immunosensor, which are discussed in the following sections.

## 5. Application of 2D MoS_2_-Based Nanocomposites

### 5.1. Electrochemical Biosensors 

Recently, significant progress has been made in the development of electrochemical sensors and their application in point-of-care diagnostics, environmental studies, food safety, drug screening, and security. Electrochemical sensing has proven to be a simple analytical technique for detection of various chemicals and biological molecules due to intrinsic advantages, such as high sensitivity and selectivity, real-time measurements, low-cost instrumentation, and the potential for miniaturized and portable devices. A standard electrochemical sensor has three components, namely, a recognition component that specifically binds the target analyte, a transducer where specific reactions occur and a signal is produced, and an electronic component that converts the obtained signal to a response [195]. The operating mechanism of an electrochemical sensor involves the interaction of the target analyte with the electrode surface coated with a catalyst, and producing the desired change in the signal as a result of a redox reaction [196]. The reaction results in electrical, thermal, or optical output signals that can be used to investigate the nature of the analyte species [197]. The active sensing materials could be biological or chemical compounds that function as catalysts for sensing specific analytes [197]. Sensitivity and selectivity are essential variables in electrochemical sensors. Thus, the surface modifications of electrodes by immobilization of specific chemical or biological recognition elements is an efficient technique used for obtaining optimal binding of the target analyte. Commonly used biorecognition elements include enzymes [69], antibodies and antigens [70,96], aptamers [95], proteins [198], etc., for high catalytic activity and excellent selectivity of the target analytes. Even so, the resulting signal from biorecognition molecules is not strong enough to achieve the ultra-sensitive detection of biomolecules required for early and rapid diagnosis of diseases [199]. Biorecognition molecules have therefore been integrated into nanomaterials to address this limitation, and to significantly improve the overall performance of the biosensor [200]. The 2D MoS_2_ nanosheets are among the nanomaterials that have become incredibly popular for sensing applications. They have been reported to be good electrode materials for electrochemical sensing; hence, there is an increasing number of publications addressing their integration into sensors [69,108]. Although 2D MoS_2_ has low electrical conductivity compared to graphene due to its large band gap limiting its use as a pristine material, 2D MoS_2_ has tunable properties that depend on the crystal structure, nanosheet size, and surface defects [146,148,161], as already discussed in detail earlier. This gives researchers the opportunity to explore the electrocatalytic properties of non-functionalized 2D MoS_2_ for the detection of biological molecules. In a study presented by Wang et al. [201], an electrochemical sensor based on non-functionalized MoS_2_ nanosheets was fabricated for detection of DNA. The reported results indicated that the bulk MoS_2_ had no electrocatalytic effect due to the low electronic conductivity that resulted from the poor interlayer electron transport. However, the exfoliated MoS_2_ nanosheets showed increased electrochemical activity with a decreased change in potential (∆Ep). The enhanced electrocatalytic activity of MoS_2_ nanosheets was discovered to be due to the anisotropic layered structure of MoS_2_ nanosheets, whose electronic structures and electrochemical activities are directly influenced by the layer thicknesses of the nanosheets [201]. 

Moreover, Sha and co-workers explored the electrocatalytic properties of hydrothermally-grown non-functionalized MoS_2_ nanosheets on aluminum foil toward non-enzymatic detection of uric acid in human urine [202]. The group demonstrated a two-step successful growth of a few layered (<4 layers) MoS_2_ nanosheets with a high ratio of 1T phase MoS_2_ than 2H phase MoS_2_. This was not a typical sensor, in the sense that the aluminum foil was used as a sensor substrate, which contributed to the excellent selectivity and reproducibility, fast response time, and low limit of detection. The impressive sensing ability also resulted from a high proportion of the metallic 1T phase MoS_2_, which provided excellent conductivity, rapid electron transfer kinetics, and more exposed catalytic active sites arising from a significant number of surface defects [202]. The results of these two sensors are based on phenomena that describe the direct structure-dependent properties of MoS_2_. Changes in the interlayer coupling, the degree of quantum confinement, and symmetry elements result in major variations in the 2D MoS_2_ electronic structure compared to bulk [9,144,145,146]. The reduction in size of MoS_2_ offers abundant exposed edges, kinks, and corner atoms that induce additional chemical effects [149,190]. Moreover, the quantum size effects lead to changes in the valence band and the oxidation potential, thereby enabling catalytic activities not possible with the bulk band structure [149].

**Figure 8:**
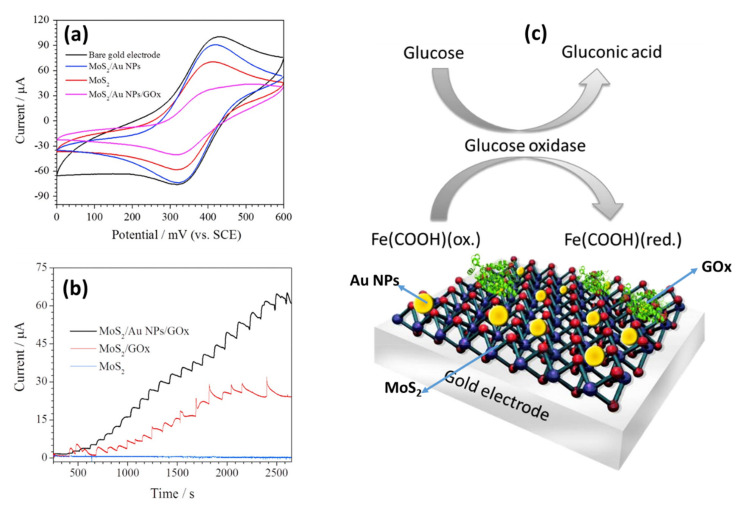
(**a**) CV response of bare and modified gold electrodes in 1 mM ferrocene carboxylic acid and 0.1 M PBS at 50 mV/s vs. Ag/AgCl reference electrode. (**b**) Amperometric responses of modified gold electrodes for the sensing of glucose concentration in the range from 0.25 to 13.2 mM at + 0.35 V applied potential. (**c**) Schematic representation of Au NPs decorated on a MoS_2_ interface and a reaction mechanism on MoS_2_-AuNPs-GOx composite [69]. Figures reproduced with permission from Ref. [69], copyright © 2016 Elsevier B.V.

Primarily, due to its fundamental drawbacks, non-functionalized 2D MoS_2_ is rarely used as both an electrocatalyst to interact directly with the target analyte and as an electron transfer mediator to enhance the signal simply because its surface area is substantially reduced. Nevertheless, the synthesis or incorporation of 2D MoS_2_ into other nanomaterials to form nanocomposites could efficiently preserve its large specific surface area and improve its catalytic activity [203]. The large surface area of 2D MoS_2_ offers effective surface functionalization and creates new possibilities for practical applications. Functionalizing 2D MoS_2_ nanosheets with metal and metal oxide NSs can effectively exploit distinct properties of both materials through a synergistic effect between metal-based NSs and MoS_2_ nanosheets, which could result in greater electrocatalytic efficiency, conductivity, and prevent restacking of nanosheets. For instance, Au NPs were post-immobilized on MoS_2_ nanosheets for electrochemical detection of glucose [69]. Electrochemical techniques, such as CV and EIS, were utilized to study the interfacial electrochemical properties resulting from the synergistic effect between Au NPs and MoS_2_ nanosheets. The CV results (Figure 8a) showed that MoS_2_ was able to promote fast electron transfer kinetics; however, a dramatic increase in the current response was observed when the electrode surface was modified with the MoS_2_-Au nanocomposite [69]. This indicates that structuring of the Au NP created a higher electroactive surface area and provided a more conductive interlayer for the transfer of electrons. In an effort to further demonstrate the efficiency of MoS_2_-Au nanocomposite, glucose oxidase (GOx) was immobilized on the MoS_2_ and MoS_2_-Au nanocomposites to achieve optimal binding of glucose and to study its electrocatalytic oxidation. According to the amperometric measurements (Figure 8b), an electrocatalytic response was achieved with both MoS_2_-GOx and MoS_2_-Au-GOx, and a steady-state current was recorded within 5 and 3 s, respectively [69]. Generally, glucose oxidase catalyzes the oxidation of glucose to produce gluconic acid and hydrogen peroxide in the presence of oxygen, as shown in Figure 8c. During this conversion, two protons and two electrons transfer from glucose to the flavin moiety of GOx [204]. While both electrodes were able to catalyze glucose, MoS_2_-Au-GOx provided good electrocatalytic performance, high sensitivity, and a low detection limit, thereby proving that the Au NPs on the surface of MoS_2_ enhanced the conductivity, improved the surface area for effective GOx immobilization, and facilitated rapid electron transfer. Although GOx is electroactive and has active sites that can be electrochemically reduced by direct electron transfer [205], it has disadvantages, such as thermal and chemical instability, critical operating conditions, such as optimum temperature, humidity and pH, low sensitivity, and reproducibility [35,93]. Controlling the interactions of enzymes with the substrate to improve electron transfer processes remains a challenge that causes a substantial loss of GOx activity, leading to poor overall performance of the sensor. Therefore, other researchers saw an opportunity to explore various 2D MoS_2_/metal-NS composites (Ni-MoS_2_ [35], CuO-MoS_2_ [82], Cu-MoS_2_ [85], Au-Pd/MoS_2_ [89], Cu_2_O-MoS_2_ [93], NiO-MoS_2_ [103], NiCo_2_O_4_-MoS_2_ [108], and MoS_2_-PPY-Au [206]) as electrode materials to construct non-enzymatic sensors due to their extraordinary electrocatalytic activities, excellent conductivity, and large surface areas. The experimental results revealed that the performances of all these 2D MoS_2_/metal-NS composites were remarkable, as shown in Table 2. These sensors demonstrated excellent electrocatalytic capabilities to oxidize glucose without the enzyme, with high sensitivity and low detection limits.

**Figure 9:**
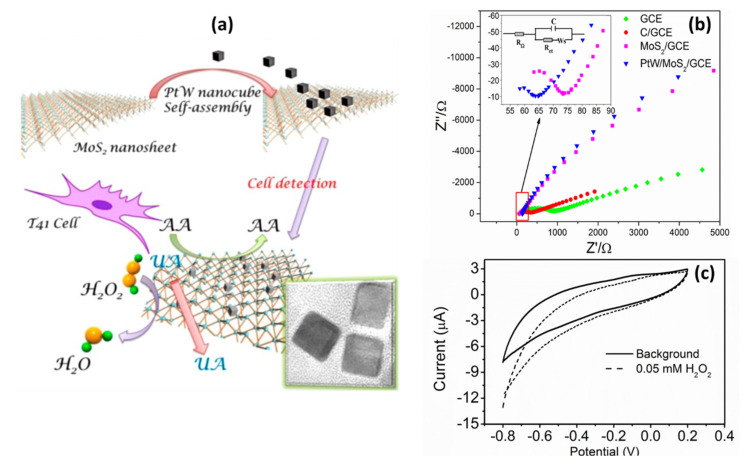
(**a**) Schematic representation of the PtW-MoS_2_ nanocomposite sensor for the detection of H_2_O_2_ released from living cells (AA: ascorbic acid, UA: uric acid). (**b**) EIS response of bare and modified electrodes in a solution containing 0.1 M KCl and 2 mM Fe(CN)_6_^3−^-Fe(CN)_6_^4−^. The equivalent circuit used to fit the Nyquist plots obtained from modified electrodes is shown in inset. (**c**) CV responses of PtW-MoS_2_ nanocomposite in the absence and presence of 0.05 mM H_2_O_2_ in 0.1 M PBS (pH = 7.4) [94]. Figures reproduced with permission from Ref. [94], copyright © 2016 Elsevier B.V.

In another study, Zhu and co-workers functionalized MoS_2_ with PtW nanocubes for detection of H_2_O_2_ released from breast cancer 4T1 cells [94] (Figure 9a). The study revealed that the functionalization of MoS_2_ with PtW nanocubes to form a composite improved the selective interaction of hydrogen peroxide (H_2_O_2_) with the sensing film, and further increased the sensitivity and selectivity of a sensor. The experimental results obtained from EIS and CV (Figure 9b,c) showed that the MoS_2_-PtW nanocomposite could catalyze H_2_O_2_ and reduce the charge transfer resistance, enabling rapid electron transfer kinetics compared to MoS_2_ alone. The group reported that the good electrocatalytic performance could be due to the fact that the integration of PtW nanocubes on the surface of MoS_2_ opened interlayer spacing in the hybrid structure, providing rich reactive sites and favorable surface permeabilities, further enhancing sensitivity during sensing processes [94]. In this sense, it is worth mentioning that the morphological structure of the PtW also played a significant role in electrocatalytic efficacy of the nanocomposite. Although the group did not study the effects of structural morphology, size, and shape toward the electrocatalytic properties of NSs, studies have shown that the structural morphologies of nanomaterials on the electrode surfaces have the potential to enhance the biomolecule sensing efficiency in terms of (i) electron transport durability, (ii) molecular adsorption capability, (iii) suitable lodging of targeted biomolecule [205,207].

Electrode modification with 2D-MoS_2_/metal-NSs composites has shown great potential in electrochemical biosensors. Current studies have demonstrated the ability of 2D-MoS_2_/metal-NSs composites to provide a convenient platform for the immobilization of biorecognition molecules and further enhance the electron transfer, resulting in faster reaction times and higher sensitivity [200]. It is therefore considered that nanocomposites based on 2D-MoS_2_/metal-NSs hold significant potential as active electrocatalysts for the development of highly sensitive and selective electrochemical sensors. Table 2 shows the performance of electrochemical biosensors based on 2D-MoS_2_/metal-NSs composites with and without biorecognition molecules for detection of various analytes. 

### 5.2. Electrochemical Immunosensors

**Figure 10:**
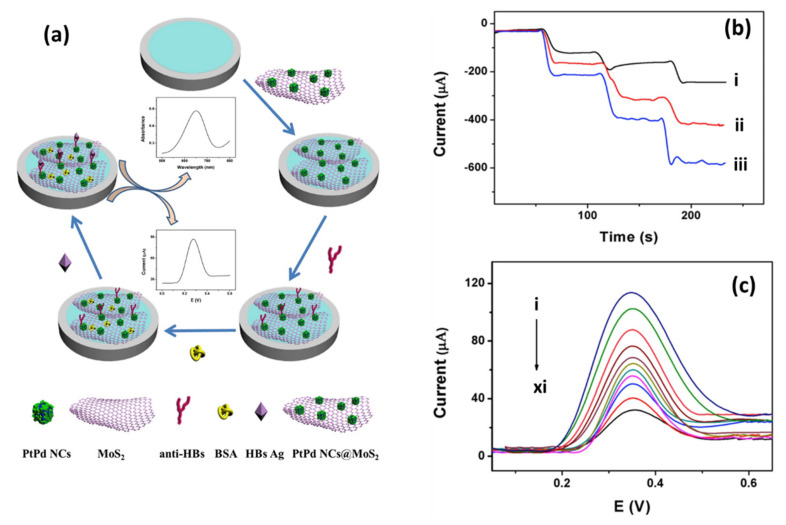
(**a**) Schematic representation of PtPd-MoS_2_ for the detection of HBs Ag and signal response mechanism. (**b**) Amperometric response: (curve i) MoS_2_, (curve ii) PtPd NCs, (curve iii) PtPd-MoS_2_ in 10 mL PBS (pH = 7.38). (**c**) DPV responses of different concentrations of HBs Ag ranging from (curve i) 32 fg/mL to (curve xi) 100 ng/mL [96]. Figures reproduced with permission from Ref. [96], copyright © 2019 Elsevier B.V.

Electrochemical immunosensors (EIs) have received considerable attention due to their high sensitivity, low cost, simple instrumentation and operation, and inherent miniaturization [214,215,216]. They show great potential in the next-generation of point-of-care (POC) diagnostics for early detection and monitoring of diseases. An electrochemical immunosensor is a type of biosensor used to detect the specific antigen–antibody recognition; it quantitatively measures the resulting electrochemical signal [70,107]. The basic immunosensor concept is to load the antibody onto the electrode surface. Following the specific antigen–antibody binding, the electron transfer rate between the electrode surface and the solution interface changes, resulting in the production of membrane potential and change of the current response, which directly reflects the concentration of the targeted antigen [107]. This type of immunoassay sandwich is a common format of an immunosensor. Countless efforts have been made into the advancements of EIs to improve performance by achieving high sensitivity. This includes the use of different nanomaterials, such as layered nanomaterials [217], metal and metal oxides NSs [218,219], and carbon-based nanomaterials [123] to amplify the EI signals. Due to excellent catalytic activity and biocompatibility, MoS_2_ functionalized with metal or metal oxide NSs has recently been introduced among nanocomposites used in the construction of immunosensors. Tan et al. [90] developed a label-free PtPd-MoS_2_ nanocomposite-based immunosensor (Figure 10a) for quantitative detection of the hepatitis B surface antigen (HBs Ag). Under optimal conditions, the PtPd-MoS_2_-based electrochemical sensor was able to attain a lower detection limit of 10.2 fg/mL compared to the colorimetric method (3.3 pg/mL). This excellent detection limit was attributed to the synergistic effect between the two nanomaterials, which resulted in an increased substrate reaction rate, electron transfer efficiency, and enhanced sensitivity. The chronoamperometry method was used to characterize the catalytic efficiency of the presented nanomaterials. The findings (Figure 10b) indicated that the PtPd-MoS_2_ nanocomposite (curve iii) produced a higher current response than MoS_2_ (curve i) and PtPd nanocubes (curve ii) due to the distinctive cubic stacking structure of PtPd on the large surface area of MoS_2_. Most importantly, the group noted that the cubic structure of PtPd offered more active sites than nanospheres, and improved the biocompatibility and the easy binding of antibodies by Pt–N bonds and Pd–N bonds, which more likely increased the amount of the bound antibody. To further demonstrate the excellent sensitivity of the prepared electrochemical immunosensors, the group used the PtPd-MoS_2_ nanocomposite in colorimetric sensors for comparison. They found that in the colorimetric sensor, the immune composite film formed on the sensing surface as the antigen concentration increased and actively inhibited the diffusion pathway of the substrate to the signal interface, resulting in a reduced current response. On the contrary, the PtPd-MoS_2_ nanocomposite exhibited excellent electrocatalytic behavior in differential pulse voltammetry (DPV) response (Figure 10c) due to the effective electron transfer kinetics [96]. The sensor was able to capture biomolecules efficiently and amplify current signals; it showed good specificity, reproducibility, and stability.

**Figure 11:**
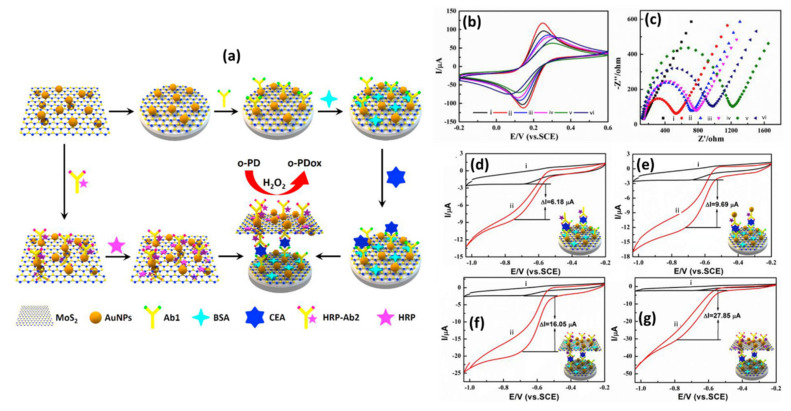
(**a**) Schematic representation of MoS_2_-Au-based immunosensor for CEA detection and signal response mechanism. (**b**) CV and (**c**) EIS curves of (i) bare GCE, modified GCE (ii) MoS_2_-Au, (iii) anti-CEA/MoS_2_-Au, (iv) BSA/anti-CEA/MoS_2_-Au, (v) CEA/BSA/anti-CEA/MoS_2_-Au and (vi) CEA/BSA/anti-CEA/MoS_2_-Au by using [Fe(CN)_6_]^3-/4-^ as an electrochemical indicator, respectively. CV responses of the proposed “sandwich” immunosensor formation with different electrodes (**d**) HRP-anti-CEA, (**e**) HRP-anti-CEA/Au, (**f)** BSA/HRP-anti-CEA/MoS_2_-Au, and (**g**) HRP/HRP-anti-CEA/MoS_2_-Au in 0.1 M PBS (i) in the absence and (ii) presence of 0.01 M o-PD + 0.16 M H_2_O_2_ [220]. Figures reproduced with permission from Ref. [220], copyright © 2019 Elsevier B.V.

The application of enzyme amplification has also been an effective way to further enhance the detection efficiency of nanomaterials. With the aim of improving detection accuracy of electrochemical immunosensors, Su and co-workers [220] developed an enzyme-assisted signal amplification approach for the detection of carcinoembryonic antigen (CEA) using the benefits of the MoS_2_-Au NPs composite and the catalytic effect of biological enzymes, as shown in Figure 11a. They immobilized horseradish peroxidase (HRP)-labeled carcinoembryonic monoclonal antibody (anti-CEA) and HRP on the MoS_2_-Au surface to create a MoS_2_-based sensing film that could greatly enhance the electrochemical response. As expected, the experimental results obtained from CV and EIS (Figure 11b,c) confirmed that the MoS_2_-Au nanocomposite had excellent electron transfer kinetics and reduced charge transfer resistance prior to enzyme immobilization. A decrease in the current response after modification with anti-CEA and HRP suggests an effective immobilization of the enzymes on the surface of MoS_2_-Au. It is commonly known that enzymes frequently inhibit the transfer of electrons and, therefore, an electroactive mediator (e.g., nanomaterials) is often required to promote the transfer of electrons. With that said, the HRP/HRP-anti-CEA/MoS_2_-AuNPs-modified electrodes showed the best electrocatalytic performance compared to other modified electrodes when they reacted with 1 ng mL^−1^ CEA in the absence (curve i) and presence (curve ii) of hydrogen peroxide H_2_O_2_ (Figure 11d–g). The excellent electrochemical response of the proposed sensors (HRP/HRP-anti-CEA/MoS_2_-AuNPs) was based on the perception that MoS_2_-Au nanocomposites improved the conductivity and increased the loading of anti-CEA and HRP-anti-CEA due to the large surface area. Moreover, The HRP labeled anti-CEA catalyzed the o-phenylenediamine (o-PD) in the presence of H_2_O_2_ and further enhanced the detection signal. Ultimately, the HRP blocked the nonspecific adsorption of the immunosensor, which multiplied the electrochemical signal due to an enzymatical catalytic reaction [220]. As a result, the fabricated immunosensor displayed high sensitivity and selectivity, good stability, and a low detection limit of 1.2 fg mL^−1^. It must be noted that, despite extensive handling and complexity of the enzymes, many researchers still find them to be ideal biorecognition molecules to improve the electrocatalytic behaviors of nano-based electrochemical sensors. Table 3 summarizes the performance of EIs based on 2D-MoS_2_/metal-NSs composites.

### 5.3. Photoelectrochemical Sensors

**Figure 12:**
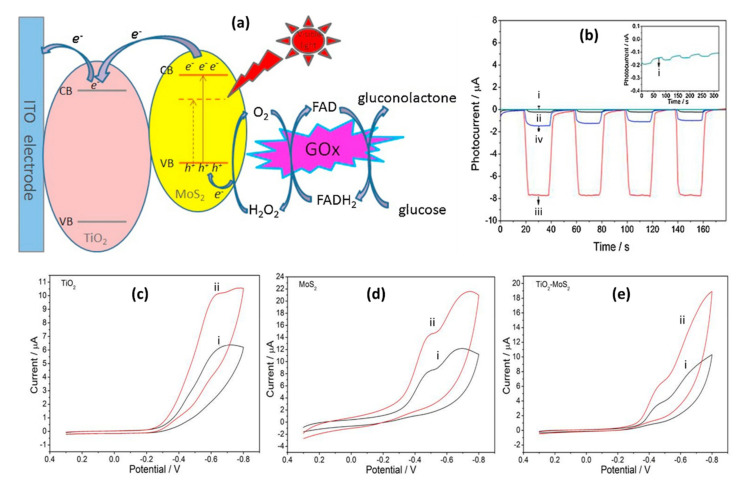
(**a**) Schematic representation of MoS_2_-TiO_2_-GOxǀITO PEC biosensor, the energy diagram and the proposed charge transfer mechanism. (**b**) Photocurrent response of (i) Pure MoS_2_|ITO, (ii) TiO_2_ nanorods|ITO, (iii) MoS_2_-TiO_2_|ITO, (iv) MoS_2_-TiO_2_-GOx|ITO in PBS solution (pH 7.4). CV of (**c**) TiO_2_-GOx, (**d**) MoS_2_-Gox, and (**e**) MoS_2_-TiO_2_-GOx (i) without and (ii) with illumination in PBS solution containing 2 mM glucose [106]. Figures reproduced with permission from Ref. [106], copyright © 2017 Published by Elsevier, Ltd.

PEC sensing has experienced rapid growth as an important branch of electrochemical detection. PEC analysis, due to high sensitivity, fast detection, and low background current, has been wildly adopted in bioanalytical chemistry as a newly emerging and continuously developing analytical method [176]. The standard PEC sensor uses light as an excitation source and photocurrent as a signal identifier. Photoactive materials are immobilized on the electrode surface as a photo-to-current converter to generate photocurrent signals under light irradiation. The photo-to-current conversion results from the electron excitation and subsequent charge transfer of a material after absorbing photons [172,226]. The total separation of the source of excitation (light) and detection signal (photocurrent) enables this method to have the advantages of a low background signal and high sensitivity to recognize target analytes in complex samples as opposed to traditional optical methods [174,227]. Photoactive materials play critical roles in PEC systems; The PEC sensor output depends on the properties of these materials since they can influence the sensitivity and selectivity of the PEC sensor. Different photoactive materials, such as metal-based NPs [227,228], quantum dots (QDs) [229], carbon-based nanomaterials [230], layered nanomaterials [176], etc., have been studied in PEC application to fulfil specific demands. Furthermore, biological molecules, such as enzymes, antibodies, nucleic acids, etc., are also important for specific recognition in PEC biosensors and immunosensors. Amongst the photoactive materials, 2D MoS_2_ is the latest emerging material for use in PEC biosensors and immunosensors because of its special optical properties. Hun et al. [176] developed a PEC sensor based on a single-layer MoS_2_ nanosheet for detection of dopamine (DA). The fabricated sensor showed an incredible photocurrent response, which demonstrated that PEC activity was enhanced by the single-layer MoS_2_. That is because single-layer MoS_2_ exhibits exceptional semiconducting electronic properties as well as a good photocurrent with a sensitive photoresponse compared to multilayer MoS_2_. The photocurrent proportionally increased with the concentration of DA in the range of 0.01 nM to 10 µM with a detection limit of 0.0023 nM. Even though different photoactive materials are available for the fabrication of PEC sensors, due to the limitations of a single material, it is difficult to achieve the ever-increasing demands for detection. However, any drawbacks of the pristine photoactive material, such as MoS_2_, can be resolved by flexible nanocomposites with the tailor-made structure and composition [173]. Additionally, certain biological molecules, such as cells and DNA, can produce photocurrents by themselves. Thus, the interactions between these biomolecules and other photoactive materials can be examined through the change of photocurrents [172]. In general, PEC biosensing or immunosensing refers to the effect on the photocurrent signal of the interaction between the biorecognition molecule and the analyte, which involves the charge and energy transfer of the PEC reaction between the electron donor/acceptor and the photoactive material during light irradiation [174]. Liu et al. [106] fabricated a PEC biosensor by immobilizing glucose oxidase on an MoS_2_ nanosheet-TiO_2_ nanorod composite modified indium tin oxide (ITO) electrode for detection of glucose (Figure 12a). The PEC properties of nanomaterials were studied by irradiating the modified electrodes with visible incident light. According to the photocurrent results (Figure 12b), both pristine MoS_2_ (curve i) and TiO_2_ (curve ii) had small photocurrent responses. The small photocurrent response of non-functionalized MoS_2_ might be attributed to poor excitation capability caused by the material’s band gap. As expected, after functionalization with TiO_2_ nanorods, the MoS_2_-TiO_2_ nanocomposite (curve iii) exhibited a photocurrent that was 4.8 times greater than that of pristine MoS_2_ and TiO_2_. It is widely understood that the interfacial interaction between MoS_2_ nanosheets and TiO_2_ nanorods and the matched energy band levels improved the visible light absorption and increased the charge separation [179]. A sudden decrease in photocurrent response was observed after immobilization of the enzyme on the MoS_2_-TiO_2_ nanocomposite (curve iv). This decrease was due to the steric obstruction of the enzyme molecules, which prevented the transport of photogenerated electrons to the electrode surface and, thus, increased their recombinations with the holes [106]. Besides the adverse effect on the photocurrent response—GOx facilitated the catalytic oxidation of glucose and improved the sensitivity the PEC sensor. The biocatalytic results obtained from CV (Figure 12c–e) indicated that the excitation of visible light enhanced the current response of the Gox-modified electrodes towards glucose, suggesting that the catalytic behavior of GOx to glucose under illumination improved the photo-electrocatalytic activity of the sensor.

The proposed mechanism (Figure 12a) of the sensor was that the lower conduction band (CB) energy level of bulk or multi-layer MoS_2_ (1.2 eV), as shown by the dash line, would prohibit the electron injection from MoS_2_ CB to the higher CB of TiO_2_. However, due to the quantum confinement effect, the energy band gap of MoS_2_ (1.8 eV) increases with the decreasing number of layers. Moreover, according to theoretical calculations, the thickness of MoS_2_ would mainly influence the conductive band (CB) level of MoS_2_, and not affect the valence band level (VB). Therefore, the CB energy level of few-layer MoS_2_ becomes higher than that of TiO_2_ (solid-line arrows), resulting in the matched energy bands between MoS_2_ and TiO_2_ and an electron injection from MoS_2_ to TiO_2_ [106,179]. When GOx is immobilized, it catalyzes the oxidization of glucose to gluconolactone, while O_2_ simultaneously reduces to H_2_O_2_ [231]. The radiation of visible-light causes the photogenerated electrons from the VB of MoS_2_ to jump to the CB of MoS_2_; this electron is transmitted to the CB of TiO_2_ and is eventually attracted to the positive ITO electrode, leaving the holes in the VB of MoS_2_. As a result, the photogenerated electron–hole pairs are effectively separated and the visible-light photoactivity is enhanced. Simultaneously, the photogenerated holes left in the VB of MoS_2_ are consumed by H_2_O_2_, which hinders the recombination of photogenerated charges and, therefore, increases the photo-current [106,179].

Inspired by the good photocatalytic properties of the MoS_2_-TiO_2_ nanocomposite, Liu and co-workers also developed a PEC aptasensor based on the TiO_2_-MoS_2_-AuNPs composite for detection of kanamycin [90]. The incorporation of Au NPs to MoS_2_-TiO_2_ was intended to further enhance the photocurrent response of the sensor. The group noticed that due to the surface plasmon resonance effect of Au NPs, the NPs would directly convert the incident visible light into electrical energy by injecting photogenerated electrons into the MoS_2_ conduction band, which would then increase the efficiency of the photoelectric conversion. As expected, the TiO_2_-MoS_2_-AuNPs composite showed an approximate two-fold increase in the photocurrent compared to the MoS_2_-TiO_2_-modified electrode. A similar trend was observed in comparison with Figure 12b (curve iv), in that, following the immobilization of the biorecognition element (aptamer), the photocurrent response of the TiO_2_-MoS_2_-Au nanocomposite decreased significantly. This was due to the steric hindrance of the biological molecules, which prevented the transport of the photogenerated electrons from moving to the electrode surface. Interestingly, under optimal conditions, the specific interaction between the aptamer and kanamycin resulted in an increase in the photocurrent response due to the oxidation of kanamycin by the photogenerated holes, which improved the overall performance of the sensor [90].

**Figure 13:**
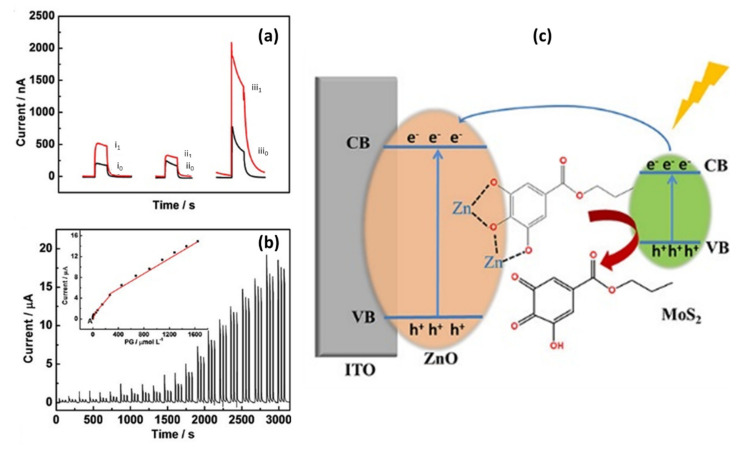
(**a**) Photocurrent responses of ZnO (curves i_0_ and i_1_), MoS_2_ (curves ii_0_ and ii_1_), and MoS_2_-ZnO (curves iii_0_ and iii_1_)-modified ITO electrodes in the absence of (curves i_0_, ii_0_, and iii_0_) and presence of (curves i_1_, ii_1_, and iii_1_) 12.43 μmol L^−1^ PG. (**b**) Photocurrent responses of MoS_2_-ZnO-modified ITO electrodes upon different concentrations of PG. The inset is the corresponding linear calibration curve. (**c**) Proposed mechanism of the MoS_2_-ZnO-based PEC sensor for the detection of PG [232]. Figures reproduced with permission from Ref. [232], copyright © 2019, American Chemical Society.

In another study, Han et al. [232] utilized the good PEC properties of the MoS_2_-ZnO nanocomposite to construct the PEC sensor for the detection of the propyl gallate (PG) antioxidant without the biorecognition element. The photoelectrochemical performance of the MoS_2_-ZnO heterostructure was studied by irradiating the modified electrodes with 470 nm of visible light. The obtained photocurrent results (Figure 13a) indicated that MoS_2_-ZnO had a higher photocurrent response in the PBS solution as compared to pristine n-type ZnO and p-type MoS_2_ nanomaterials. A similar trend was observed upon addition of 12.43 μmol L^−1^ PG; MoS_2_-ZnO showed a drastic increase in the photocurrent response compared to pristine ZnO and MoS_2_. The group indicated that the incorporation of ZnO into MoS_2_ tends to form a p−n heterojunction for efficient carrier separation and light absorption, leading to a high photocurrent response. Furthermore, the photocurrent response of the MoS_2_-ZnO-modified electrode to a range of PG concentrations was analyzed and the results demonstrated a favorable linear relationship between the wide range of PG concentrations (0.1249–1643 μmol L^−1^) and the photocurrent responses, as shown in Figure 13b. The sensor displayed outstanding PG detection capability with undeniably higher sensitivity and selectivity, good stability, and a wider linear range. According to the proposed mechanism (Figure 13c), the three adjacent PG hydroxyl groups bind to the Zn(II) surface site of ZnO to form a chelate structure, resulting in a higher concentration of PG near the MoS_2_-ZnO photocatalyst. Thereafter, the electrons and holes of MoS_2_-ZnO separate and generate a photocurrent under light irradiation. The electrons would then be transferred from MoS_2_ to ZnO and, in response, the holes formed by MoS_2_ would efficiently oxidize PG. The proper conduction band formed in MoS_2_-ZnO nanocomposites effectively enhances the transfer of electrons from ZnO to the ITO electrode, which not only decreases the recombination rate between the electron and the hole, but also enhances the sensitivity of the MoS_2_-ZnO-based PEC sensor. Table 4 summarizes the performances of various PEC sensors based on 2D-MoS_2_/metal-NSs composites [232].

### 5.4. Electrochemiluminescence Sensors

ECL detection is commonly used in the various applications for biosensors. This technique combines the advantages of electrochemistry and chemiluminescence in resolving single sensing limitations, making it more desirable for precise detection and enhancement of a sensor’s sensitivity and specificity. ECL refers to the light emission from an excited state produced by the electron transfer reaction between species at electrode surfaces following electrochemical reactions [236]. ECL detection consists of measuring photon output and, thus, the light intensity emitted in the solution during an electrochemical reaction. The light intensity is therefore directly proportional to the concentration of one or more of the reactants involved in the electrochemical reaction [237]. ECL has many advantages over photoluminescence, such as simplicity, high sensitivity, and rapidity response, and does not require the use of an external light source, leading to a high luminescent purity and a low optical background noise [236,238]. Furthermore, the ECL emission light can be initiated and controlled with high reproducibility and accuracy by alternating the applied potential [236,239]. Unfortunately, one of the drawbacks of the ECL method is the possibility of electrode fouling, which can result in poor reproducibility. While this could sometimes be a concern in the presence of complex samples, this effect can be avoided by frequent electrochemical cleaning of the electrodes. Moreover, new electrode materials that are less prone to fouling, such as boron-doped diamond and disposable screen-printed electrodes, have been introduced and have become more popular [240]. The continuous development of nanomaterials offers new opportunities for conventional ECL research, mainly owing to their large surface area, abundant active sites, and distinctive optical properties. Nanomaterials with good electron transfer capabilities, such as 2D nanomaterials [241], quantum dots [242], and metal nanoparticles [243], have also been implemented to improve the efficiency of ECL sensors. Among these nanomaterials, the use of 2D MoS_2_ in ECL sensors is not so prominent. This is attributable to the fact that 2D MoS_2_ is not capable of generating electrochemiluminescence; however, it serves other functions, such as acting as a robust substrate or amplifying the signal [244]. MoS_2_ can also be used as a carrier for other nanomaterials with structural and compositional benefits to improve the ECL performance. 

**Figure 14:**
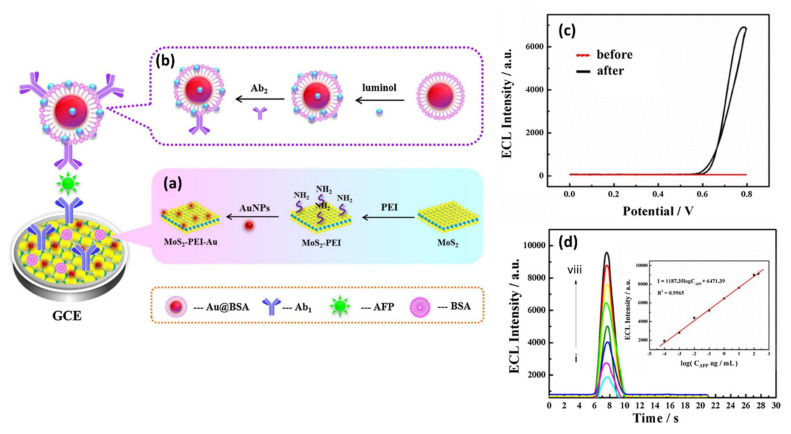
Schematic representation of the proposed immunosensor. (**a**) Formation of MoS_2_-PEI-Au nanocomposites and (**b**) the preparation procedure of the luminol-Au@BSA-Ab2 bioconjugation. (**c**) ECL profiles of the immunosensor before and after incubation with luminol-Au@BSA-Ab2 bioconjugate in 0.01 M pH 7.4 PBS containing 3 mM H_2_O_2_ and 10 ng/mL AFP. (**d**) ECL responses for AFP detection. The concentrations of AFP (ng/mL): (i) 0.0001, (ii) 0.001, (iii) 0.01, (iv) 0.1, (v) 1, (vi) 10, (vii) 100, and (viii) 200 (inset: calibration curve) [81]. Figures reproduced with permission from Ref., copyright © 2017 Elsevier B.V.

In that context, Zhang et al. [81] fabricated a sandwich ECL immunosensor for the detection of alpha fetal protein (AFP) based on MoS_2_-PEI-Au nanocomposites. As shown in Figure 14a, polyethylene (PEI) was first introduced to the surface of the MoS_2_ nanosheets to serve as a binding agent that can absorb the negatively charged Au NPs by electrostatic attraction. The formed MoS_2_-PEI-Au nanocomposite was used to capture the Ab_1_ and Au@BSA-luminol as a signal probe to immobilize Ab_2_, respectively (Figure 14b). In order to confirm the efficiency of the Au@BSA-luminol signal probe, the group studied the ECL behavior of the immunosensor before and after the immobilization of Au@BSA-luminol (Figure 14c) on the surface of MoS_2_-PEI-Au-Ab1 in the presence of H_2_O_2_ and AFP. The obtained result indicated that the MoS_2_-PEI-Au modified electrode could produce the ECL signal only after the presence of Au@BSA-luminol-Ab2, which demonstrated the efficiency of the bioconjugate as a luminescence reagent. In addition, the substantial enhancement on the ECL-sensing signal was due to the catalytic efficiency of MoS_2_ nanosheets, which could decompose H_2_O_2_ to increase the ECL intensity of luminol, and the excellent electrical conductivity of Au NPs, which facilitated the electron transfer and further enhanced the sensitivity of the ECL immunosensor. Under optimal conditions, a fabricated sandwiched immunoassay sensor was used to detect a series of AFP concentrations in 0.01 M PBS. The ECL intensity was found to increase simultaneously with an increase in concentration of AFP, as shown in Figure 14d. The target AFP molecules were being sandwich, captured between the primary antibody and the Au@BSA-luminol-Ab2 bioconjugate by means of a immune-specific reaction, leading to an enhanced detectable ECL signal. Based on the low detection limit obtained, the high sensitivity and increased intensity of the ECL signals, a rapid and sensitive quantitative detection of AFP was therefore successfully attained. Table 5 summarizes the performances of a few ECL sensors that have been reported based on 2D-MoS_2_/metal-NSs composites.

Focusing on the application of 2D MoS_2_-metal NS composites on various electrochemical sensors, it can be inferred that 2D MoS_2_ represents a new class of highly electroactive materials. The large surface areas of 2D MoS_2_ nanosheets enable surface functionalization with a variety of nanomaterials, providing new opportunities for 2D material-based functional devices. The effective support structure of MoS_2_ nanosheets allows for a good distribution of metal-based NSs, resulting in a synergistic effect that enhances catalytic efficiency and conductivity over a pristine MoS_2_. Furthermore, 2D-MoS_2_/metal-NS composites provide a convenient platform for the immobilization of biorecognition molecules and enhance electron transfer, resulting in faster reaction times as well as increased sensitivity and selectivity. Therefore, 2D MoS_2_-NSs composites are thought to have considerable potential as effective electrocatalysts for the production of highly responsive electrochemical sensors with low limits of detection.

## 6. Challenges, Limitations, and Future Outlooks

The fabrications of 2D MoS_2_ nanosheets as well as their use in electrochemical sensors have advanced significantly in recent years. However, uniform growth of high quality 2D MoS_2_ nanosheets in large-scale production remains a challenge. It is generally acceptable that controlling the number of layers, size, purity, stoichiometry, and phases of 2D MoS_2_ is not only enigmatic but also a demanding task. Although liquid–phase exfoliation is ideal for high scalability and crystallinity, a low yield of monolayers is inevitable, while the size distribution is rather wide. Moreover, environmental stability of 2D MoS_2_ poses some significant challenges. At ambient temperatures, pristine MoS_2_ nanosheets degrade gradually due to surface contamination and significant oxygen adsorption in an air environment [19,20]. More developments and optimization of the MoS_2_ nanosheet preparation methods are necessary to improve the quality of the material. Furthermore, emphasis should be placed on the development of improved surface functionalization methods capable of providing good control over the size and distribution of metal-based NSs on MoS_2_. It is fundamentally important to achieve a homogeneous dispersion of non-agglomerated NPs in the nanocomposite matrix using both in situ and ex situ functionalization methods. The uniform distribution of NPs degrades the properties of the nanocomposite. A high loading of NPs on the surface of MoS_2_ was also discovered to cause decay [69]. 

Metal-based NSs have shown great capability to improve the electrical, optical, and catalytic properties of 2D MoS_2_ in response to various redox processes. It is a known fact that metal-based NSs provide more active sites on the surface of the MoS_2_ nanosheet to improve electrocatalytic activity and electron transfer kinetics for the detection of various target analytes. However, because a MoS_2_-metal-based nanocomposite cannot electrocatalyze most biological molecules, the use of biological recognition elements, such as enzymes, aptamers, and antigen-antibodies as bioreceptors, remains prevalent. Extensive research on the mechanism of functionalization and its effect on the electronic band structures of MoS_2_ is lacking. As a result, failing to understand the interactions between MoS_2_ nanosheets and NPs limits the ability to further improve these nanocomposites for use in electrochemical sensors. In this sense, a thorough understanding of the catalytic mechanisms and structure configurations of MoS_2_ active sites can provide valuable guidance in resolving existing issues, potentially eliminating the need for biological recognition elements. Furthermore, stability of MoS_2_-metal-based nanocomposites was shown to be one of the limiting factors for improved catalytic efficiency. Most sensors showed a decrease in performance over a period of 4 weeks, indicating long-term stability issues of the MoS_2_-metal-based nanocomposites [96,198,212,223]. This is a problem because sensor stability and durability are critical requirements in commercial and industrial settings. Therefore, core advancements should be on developing various synthesis methods for 2D MoS_2_ nanocomposites that exhibit outstanding stability. Nonetheless, given recent advancements in the fabrication of MoS_2_-based sensors and biosensors, we anticipate extensive use of 2D-MoS_2_/metal-NSs composites for developing dimension-tailored devices, such as wearable sensors, for a variety of applications in the near future.

## 7. Conclusions

We highlighted some main features of MoS_2_ nanosheets when they are exfoliated into single or a few layers, the effects on properties when functionalized with metal and metal oxide NSs, and recent advances in electrochemical sensors based on 2D-MoS_2_/metal-NSs composites. Reducing the number of layers results in a gradual shift in the indirect band gap to a large direct band gap in single layers, which turns the material into a 2D semiconductor. Multiple methods have been suggested for synthesizing 2D MoS_2_; these methods can also be used to tune the 2D MoS_2_ electronic band structure. Methods (e.g., the Li intercalation and CVD) were found to alter the optical and electrical properties of material by simply manipulating the experimental conditions. The Li intercalation of MoS_2_ results in a structural transformation of MoS_2_ from phase 2H to phase 1T, as well as a transition from the semiconductor to metallic 2D MoS_2_ nanosheet. The interesting thing to note is, owing to the nature of the exfoliation process, many imperfections may occur on the material surface. Control of high quality, large surface areas, and a uniform 2D MoS_2_ are therefore necessary in order to further produce desirable nanosheets and to strongly understand the material’s surface chemistry. While various methods for preparing 2D MoS_2_ nanosheets have been developed, obtaining the perfect 2D MoS_2_ fitting the above requirements remains a challenge. Even so, 2D MoS_2_ and other layered materials still play important roles in the general application of sensors. On the other hand, functionalizing or doping MoS_2_ nanosheets would adjust or add the required properties to the material, which can be used in the fabrication of sensors. Integrating metal-based NSs on MoS_2_ nanosheets can effectively exploit the distinct qualities of both materials. This can be accomplished by ex situ or in situ functionalization. While it has been confirmed that MoS_2_ simply forms nanocomposites with metal and metal oxide NSs, achieving a homogeneous dispersion of non-agglomerated nanoparticles within the nanocomposite matrix remains a challenge. We noted that the method of preparation, functionalization, and layer thickness play important roles in the electrical, optical, and catalytic properties of the material. Control over these properties during production is therefore essential for the ultimate application regardless of the method of synthesis and functionalization. Recently, various sensors based on 2D-MoS_2_/metal-NSs composites have been developed, from field effect transistors (FET) to electrochemical sensors. Emerging research and publications have shown that 2D-MoS_2_/metal-NSs composites serve as good platforms for sensing. The nanocomposites offer a new range of highly-electroactive materials and may further advance to better the electrochemical sensing performance.

## Figures and Tables

**Table 1:** Functionalization methods of MoS_2_ nanosheets with different metal and metal oxide NSs and their applications in electrochemical sensors.

	MoS_2_-NS Composites	Method of Functionalization	Metal and Metal Oxides Structural Morphology	Size (Diameter)	Type of Sensor	Ref.
Ex situ functionalization	MoS_2_-Au	Post immobilization	Nanoparticles	5 nm	Electrochemical biosensor	[69]
	TTR-MoS_2_-Au	Post immobilization	Nanocrystals	-	Photoelectrochemical immunosensor	[80]
	MoS_2_-PEI-Au	Post immobilization	Nanoparticles	12 nm	Electrochemiluminescence immunosensor	[81]
	CuO-MoS_2_	Post immobilization	Nanotubes	20 nm	Electrochemical sensor	[82]
In situ functionalization	MoS_2_-Au	Electrodeposition	Nanoparticles	10 m	Electrochemical aptasensor	[13]
	MoS_2_-MWCNT/Au	Electrodeposition	Nanoparticles	3–5 nm	Electrochemical sensor	[83]
	MoS_2_-Au/Pt	Electrodeposition	Nanoparticles	100 nm	Electrochemical biosensor	[84]
	Cu-MoS_2_	Electrodeposition	Nanoflowers	-	Electrochemical biosensor	[85]
	ZnO-MoS_2_	Electrodeposition	Nanosheets	50 nm	Electrochemical sensor	[86]
	Ni-MoS_2_-Naf	Chemical reduction	Nanoparticles	6 nm	Electrochemical sensor	[35]
	Au-MoS_2_	Chemical reduction	Nanoparticles	80 nm	Electrochemical sensor	[87]
	N/F/MoS_2_-Ag	Chemical reduction	Nanoparticles	3 nm	Electrochemical sensor	[88]
	Au-Pd/MoS_2_	Chemical reduction	Nanoparticles	-	Electrochemical sensor	[89]
	TiO_2_-MoS_2_-Au	Chemical reduction	Nanoparticles	5–10 nm	Photoelectrochemical aptasensor	[90]
	Pt-MoS_2_	Chemical reduction	Nanoparticles	2.5 nm	Electrochemical biosensor	[91]
	PtNi-MoS_2_	Chemical reduction	Nanoparticles	1.35–6.26 nm	Electrochemical sensor	[92]
	Cu_2_O-MoS_2_	Chemical reduction	Nanoparticles	6–18 nm	Electrochemical sensor	[93]
	PtW-MoS_2_	Chemical reduction	Nanocubes	10 nm	Electrochemical sensor	[94]
	Pd–MoS_2_	Chemical reduction	Nanoparticles	-	Electrochemical aptasensor	[95]
	PtPd-MoS_2_	Chemical reduction	Nanocubes	50 nm	Electrochemical immunosensor	[96]
	Pt-MoS_2_	Chemical reduction	Nanoparticles	-	Electrochemical sensor	[97]
	Ag-MoS_2_	Chemical reduction	Nanoparticles	5 nm	Electrochemical sensor	[98]
	MoS_2_-Pt	Chemical reduction	Clover-like nanoparticles	15.3–2 nm	Electrochemical sensor	[99]
	Au−Pd−Pt/MoS_2_	Chemical reduction	Nanoflowers	14–26 nm	Electrochemical sensor	[100]
	PdNi-MoS_2_	Chemical reduction	Nanowires	0.5–3 nm	Electrochemical sensor	[101]
	MoS_2_-Cu_2_O-Au	Hydrothermal reaction	Nanocrystals	20–30 nm	Electrochemical immunosensor	[102]
	NiO-MoS_2_	Hydrothermal reaction	Nanoparticles	38–72 nm	Electrochemical sensor	[103]
	Fe_2_O_3_-MoS_2_	Hydrothermal reaction	Nanoflowers	-	Electrochemical sensor	[104]
	Fe_3_O_4_-MoS_2_	Hydrothermal reaction	Nanospheres	20–30 nm	Electrochemical sensor	[105]
	MoS_2_-TiO_2_	Hydrothermal reaction	Nanorods	20 nm	Photoelectrochemical biosensing	[106]
	Ag/MoS_2_@Fe_3_O_4_	Hydrothermal reaction	Nanospheres	50 nm	Electrochemical immunosensor	[107]
	MoS_2_-Cu_2_O/Pt	Solvothermal reaction	Nanoparticles	15 and 3 nm	Electrochemical immunosensor	[70]
	Cu-MoS_2_-Naf	Solvothermal reaction	Nanoparticles	1–5 nm	Electrochemical sensor	[108]
	NiCo_2_O_4_-MoS_2_	Solvothermal reaction	Nanorods	-	Electrochemical sensor	[109]

**TTR**; transthyretin, **PEI**; polyethylenimine, **CuO**; copper oxide, **MWCNT**; multiwalled carbon nanotubes, **Cu_2_O**; cuprous oxide, **Cu**; copper, **ZnO**; zinc oxide, **Naf**; Nafion, **N/F**; nitrogen fluorine, **TiO_2_**; titanium dioxide, **PtW**; platinum/tungsten, **NiO**; nickel oxide, **Fe_2_O_3_, Fe_3_O_4_**; iron (II, III) oxide, **NiCo_2_O_4_**; nickel cobaltite.

**Table 2:** Electrochemical biosensors based on 2D-MoS_2_/metal-NSs composites.

Sensor	Analyte	Electrochemical Method	Linear Range	LOD	Ref.
Au-MoS_2_/GCE	ATP Thrombin	SWV	1 nM–10 mM 0.01 nM–10 µM	0.32 nM 0.0014 nM	[13]
GCE/Ni-MoS_2_/Naf	Glucose	Amperometry	0–4 mM	0.31 M	[35]
MoS_2_/Au/GOx	Glucose	Amperometry	0.25–13.2 mM	0.042 µM	[69]
CuO/MoS_2_/GCE	Glucose	Amperometry	35–800 μM	0.017 μM	[82]
MoS_2_−Au/Pt@GCE	H_2_O_2_	Amperometry	10 μM–19.07 mM	0.39 μM	[84]
Cu-MoS_2_/GCE	H_2_O_2_ glucose	Amperometry	0.04–35.6 μM 1–70 μM	0.021 μM 0.32 μM	[85]
ZnO/MoS_2_/GCE	DNA	DPV	1.0 fM–1.0 µM	0.66 fM	[86]
Au@MoS_2_/GCE	AA DA UA	DPV	20–300 µmol/L 5–200 µmol/L 20–400 µmol/L	3.0 µmol/L 1.0 µmol/L 5.0 µmol/L	[87]
Au-Pd/MoS_2_/GCE	H_2_O_2_ Glucose	DPV Amperometry	0.8 µM–10 Mm 0.5–20 mM	0.16 µM 0.40 mM	[89]
Pt-MoS_2_/GCE	H_2_O_2_	Amperometry	0.004–48.5 mM	0.001 mM	[91]
PtNi@MoS_2_/GCE	DA UA	DPV	0.5–250 µM 0.5–1800 µM	0.1 µM 0.1 µM	[92]
Cu_2_O/MoS_2_/GCE	Glucose	Amperometry	0.01–4.0 mM	1.0 µM	[93]
PtW/MoS_2_/GCE	H_2_O_2_	Chronoamperometry	1 μM–0.2 mM	5 nM	[94]
Pd/PDDA–G–MoS_2_/GCE	TB	DPV	0.0001–40 nM	0.062 pM	[95]
PtNPs@MoS_2_/GCE	DA UA	DPV	0.5–150 μmol/L 5–1000 μmol/L	0.12 μmol/L 0.8 μmol/L	[97]
Ag@MoS_2_/GCE	DA	DPV	1−500 μM	0.2 μM	[98]
MoS_2_-CPtNPs/GCE	DA UA	DPV	5–200 µΜ 20–500 μM	0.39 μM 1.8 μM	[99]
Laminin/Au−Pd−Pt/MoS_2_/SPCE	H_2_O_2_	Amperometry	1–100 nM	0.3 nM	[100]
NiO/MoS_2_/GCE	Glucose	Amperometry	0.01–10 mM	1.62 μM	[103]
GCE/Cu-MoS_2_/Nafion	Glucose	Amperometry	0–4 mM	-	[108]
NiCo_2_O_4_-MoS_2_/chitosan/GCE	Glucose	Amperometry	0.0007–13.78 mM	0.23 µM	[109]
MoS_2_-PPY-AuNPs/GCE	Glucose	DPV	0.1–80 nM	0.08 nM	[206]
AuNPs@MoS_2_/GCE	miRNA-21	DPV	10 fM–1 nM	0.78 fM	[208]
Chox/MoS_2_-AuNPs/GCE	Cholesterol	Amperometry	0.5–48 μM	0.26 ± 0.015 μM	[209]
MoS_2_-Au-PEI-hemin	Clenbuterol (CLB)	DPV	10 ng/mL–2 μg/mL	1.92 ng/mL	[210]
NF/AuNPs/CuO-MoS_2_	Glucose	Chronoamperometry	0.5 μM–5.67 mΜ	0.5 μM	[211]
MCH/dsDNA/MoS_2_-AuNPs/GCE	T4 polynucleotide kinase (PNK)	SWV	0.001–10 U/mL	2.18 × 10^−4^ U/mL	[212]
miRNA/MCH/SH-RNA/AuNPs-MoS_2_/FTO	MicroRNA-155	DPV	1 fM–10 nM	0.32 fM	[213]

**ATP**; triphosphate, **DA**; dopamine, **DNA**; deoxyribonucleic acid, **DPV**; differential pulse voltammetry, **SWV;** square Wave Voltammetry, **PDDA–G**; poly(diallyldimethylammonium chloride)–graphene, **TB**; thrombin, **CPtNPs**; Clover-like platinum nanoparticle, **PPY**; polypyrrole, **miRNA-21**; microribonucleic acid-21, **Chox**; cholesterol oxidase, **NF**; Nafion, **MCH**; 6-mercaptohexanol, **FTO**; fluorine doped tin oxide.

**Table 3:** Electrochemical immunosensors based on 2D-MoS_2_/metal-NSs composites.

Sensor	Analyte	Electrochemical Method	Linear Range	LOD	Ref.
MoS_2_@Cu_2_O-Pt/Ab_2_	hepatitis B antigen	Amperometry	0.5 pg/mL–200 ng/mL	0.15 pg/mL	[70]
BSA/anti-HBs/PtPd NCs@MoS_2_/ GCE	Hepatitis B antigen	DPV	32 fg/mL–100 ng/mL	10.2 fg/mL	[96]
MoS_2_@Cu_2_O-Au-Ab_2_	Alpha fetoprotein (AFP)	Amperometry	0.1 pg/mL–50 ng/mL	0.037 pg/mL	[102]
Ab_2_-Ag/MoS_2_@Fe_3_O_4_/MGCE	carcinoembryonic antigen (CEA)	DPV	0.0001–20 ng/mL	0.03 pg/mL	[107]
HRP/HRP-anti-CEA/MoS_2_-AuNPs	carcinoembryonic antigen (CEA)	DPV	10 fg/mL–1 ng/mL	1.2 fg/mL	[220]
GCE/MoS_2_-Au-Ab_1_	CEA	DPV	1 pg/mL–50 ng/mL	0.27 pg/mL	[221]
Pd NPs@MoS_2_/NiCo	Procalcitonin	Chronoamperometry	0.001–50 ng/mL	0.36 pg/mL	[222]
Au-MoS_2_/ITO	Triiodothyronine (T_3_)	EIS	0.01–100 ng/mL	2.5 pg/mL	[223]
Cu-MoS_2_/GCE	3-phenoxybenzoic acid (3-PBA),	EIS	0–6 µg/mL	3.8 µM	[224]
Tac/BSA/Ab/PS-AuNRs@L-Cys-MoS_2_/GCE	Tacrolimus (Tac)	DPV	1.0–30 ng/mL	0.17 ng/mL	[225]

**Ab_1_**; primary antibody, **Ab_2_**; secondary antibody, **HBs Ag**; Hepatitis B surface antigen, **BSA**; bovine serum albumin, **ITO**; indium tin oxide, **L-Cys**; L-cysteine, **PS-AuNRs**; polystyrene-gold nanorods.

**Table 4:** Photoelectrochemical sensors based on 2D-MoS_2_/metal-NSs composites.

Sensor	Analyte	Linear Range	LOD	Ref.
TTR/AuCNs/MoS_2_/GCE	Tetrabromobisphenol A	0.1 nM−1.0 µM.	0.045 nM	[80]
BSA|aptamer|TiO_2_-MoS_2_-AuNP|ITO	kanamycin	0.2 nM−450 nM	0.05 nM	[90]
GOx|MoS_2_-TiO_2_|ITO	Glucose	0.1−10.5 mM	0.015 mM	[106]
MoS_2_-ZnO|ITO	Propyl gallate	0.1249−1643 μmol/L	1.2 × 10^−8^ mol/L	[232]
Au/MoS_2_/TiO_2_	Glucose	5−1000 μM	1.3 nM	[233]
Pro-GRP-MIP/AuNPs/2D-MoS_2_/GCE	Pro-gastrin-releasing peptide (Pro-GRP)	0.02–5 ng/mL	0.0032 ng/mL	[177]
Au-MoS_2_/FTO	anti-human IgG	41.7 nM–4.17 μM	4.17 nM	[178]
ITO/MTiO_2_-AuNPs-MoS_2_-GOx	Glucose	0.004–1.75 mM	1.2 μM	[234]
biotin DNA/MoS_2_-AuNPs/ITO	miRNA	10 fM–1 nM	4.21 fM	[235]

**MIP**; molecularly imprinted polymer.

**Table 5:** Electrochemiluminescence sensors based on 2D-MoS_2_/metal-NSs composites.

Sensor	Analyte	Linear Range	LOD	Ref.
luminol-Au@BSA-Ab_2_/AFP/BSAT/Ab_1_/Chi/MoS_2_-PEI-Au/GCE.	Alpha fetal protein (AFP),	0.0001−200.0 ng/mL	1.0 × 10^−5^ ng/mL	[81]
BSA/Ab2/ABEI-Cys/Au–Pd–Pt/MoS_2_	cystatin C (CYSC)	1.0 fg/mL−5.0 ng/mL	0.35 fg/mL	[186]
QDs–Apt2/PDGF-BB/Apt1/MoS_2_–AuNPs/GCE	platelet-derived growth factor-BB	0.01−100 pmol/L	1.1 fmol/L	[187]

**ABEI**; N-(aminobutyl)-N-(ethylisoluminol), **PDGF-BB**; platelet-derived growth factor BB, **QD**s; quantum dots.

## Data Availability

Not applicable.
